# Signaling pathways and targeted therapy for myocardial infarction

**DOI:** 10.1038/s41392-022-00925-z

**Published:** 2022-03-10

**Authors:** Qing Zhang, Lu Wang, Shiqi Wang, Hongxin Cheng, Lin Xu, Gaiqin Pei, Yang Wang, Chenying Fu, Yangfu Jiang, Chengqi He, Quan Wei

**Affiliations:** 1 Rehabilitation Medicine Center and Institute of Rehabilitation Medicine, West China Hospital, Sichuan University, Chengdu, Sichuan PR China; 2 Key Laboratory of Rehabilitation Medicine in Sichuan Province, Chengdu, Sichuan PR China; 3 National Clinical Research Center for Geriatrics, West China Hospital, Sichuan University, Chengdu, Sichuan PR China; 4 Aging and Geriatric Mechanism Laboratory, West China Hospital, Sichuan University, Chengdu, Sichuan PR China; 5 State Key Laboratory of Biotherapy, West China Hospital, Sichuan University, Chengdu, Sichuan PR China

**Keywords:** Cardiovascular diseases, Molecular medicine

## Abstract

Although the treatment of myocardial infarction (MI) has improved considerably, it is still a worldwide disease with high morbidity and high mortality. Whilst there is still a long way to go for discovering ideal treatments, therapeutic strategies committed to cardioprotection and cardiac repair following cardiac ischemia are emerging. Evidence of pathological characteristics in MI illustrates cell signaling pathways that participate in the survival, proliferation, apoptosis, autophagy of cardiomyocytes, endothelial cells, fibroblasts, monocytes, and stem cells. These signaling pathways include the key players in inflammation response, e.g., NLRP3/caspase-1 and TLR4/MyD88/NF-κB; the crucial mediators in oxidative stress and apoptosis, for instance, Notch, Hippo/YAP, RhoA/ROCK, Nrf2/HO-1, and Sonic hedgehog; the controller of myocardial fibrosis such as TGF-β/SMADs and Wnt/β-catenin; and the main regulator of angiogenesis, PI3K/Akt, MAPK, JAK/STAT, Sonic hedgehog, etc. Since signaling pathways play an important role in administering the process of MI, aiming at targeting these aberrant signaling pathways and improving the pathological manifestations in MI is indispensable and promising. Hence, drug therapy, gene therapy, protein therapy, cell therapy, and exosome therapy have been emerging and are known as novel therapies. In this review, we summarize the therapeutic strategies for MI by regulating these associated pathways, which contribute to inhibiting cardiomyocytes death, attenuating inflammation, enhancing angiogenesis, etc. so as to repair and re-functionalize damaged hearts.

## Introduction

Cardiovascular diseases are the leading cause of death disease worldwide, of which the death toll due to ischemic heart disease accounted for as much as 49.2% in 2019^1,2^. Acute myocardial infarction (MI) is usually caused by a thrombus blocking an artery or a bypass graft, characterized by an abrupt reduction in blood flow to the myocardium, ultimately leading to heart failure and death^2,3^. Restoring blood flow to rescue hypoxic-ischemic tissue is considered to be an effective strategy^4–6^. Thrombolysis, percutaneous coronary intervention (PCI), and coronary artery bypass grafting are the most common methods for the treatment of acute MI in the clinic^4–6^. Although these methods significantly reduce the patient mortality rate^7^, complications occur in an unpredictable manner, including hemorrhage, ischemia-reperfusion injury, and coronary restenosis^5,8^. Therefore, it is necessary to pursue more innovative and effective avenues to preserve myocardial function and avoid heart failure progression.

**Fig. 1:**
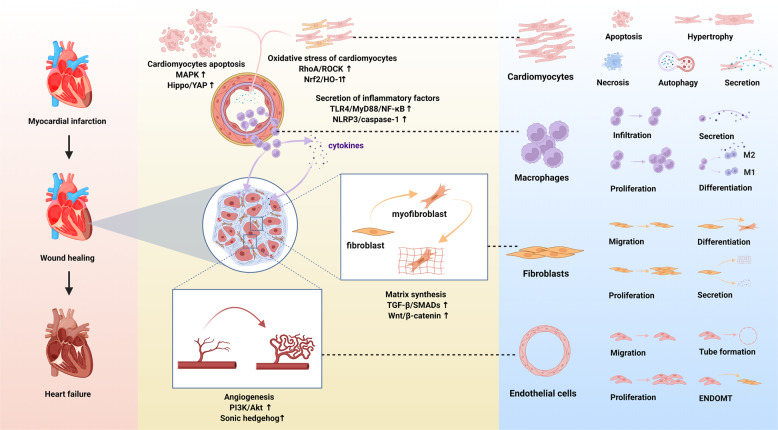
Schematic diagram of the pathophysiology of different cell phenotypes and representative pathways involved in infarct hearts (created with BioRender.com). After myocardial infarction, various cell signaling pathways are activated. Oxidative stress and the death of tissue, particularly apoptotic and necrotic cardiomyocytes, trigger the inflammatory response. Immunocytes infiltrate the infarct area and release inflammatory factors. Meanwhile, cardiac fibroblasts transform into cardiac myofibroblasts and secrete extracellular matrix, and endothelial cells migrate, proliferate and form a network of blood vessels to promote the cardiac repair. However, pathological hypertrophy of the myocardium affected by inflammation, coupled with reactive fibrosis, would eventually lead to cardiac remodeling and heart failure. MAPK, mitogen-activated protein kinase; Hippo/YAP, Hippo/Yes-associated protein; RhoA/ROCK, Ras homolog family member A/Rho associated coiled-coil containing protein kinase; Nrf2/HO-1, nuclear factor erythroid derived 2-related factor 2/heme oxygenase-1; TLR4/MyD88/NF-κB Toll-like receptor 4/MyD88/nuclear factor-κB; NLRP3/caspase-1, the nucleotide-binding domain, leucine-rich-repeat family, pyrin-domain-containing 3/caspase-1; TGF-β/SMADs, transforming growth factor-β/SMADs; Wnt/β-catenin, Wingless/β-catenin; PI3K/Akt, phosphoinositide-3 kinase/protein kinase B; EndoMT. endothelial-to-mesenchymal transition

Post MI, in the injured myocardium, the inflammation, fibrosis, and angiogenesis phases in the injured myocardium overlap^9,10^ (Fig. 1). Suffering from ischemia-hypoxia, the apoptotic wave of cardiomyocytes within hours to days, and the damaged tissue triggers an inflammatory reaction, which results in the development of granulation tissue with infiltration of immunocytes that release pro-inflammatory cytokines and chemokines^9,11^. Along with the recruitment of myeloid cells and the transduction of pro-inflammatory signals, including transforming growth factor-β (TGF-β)/SMADs and Wingless (Wnt)/β-catenin, fibroblasts produce collagen and endothelial cells are activated by pro-angiogenic phosphoinositide-3 kinase/protein kinase B (PI3K/Akt), Janus kinase/signal transducer and activator of transcription (JAK/STAT), and angiogenesis commences^9–12^. The new capillaries not only bring nutrients to the border zone of the infarct but also provide energy for fibroblasts to differentiate into myofibroblasts, which is crucial for sustaining the integrity of the structure and function of the heart through compensation^9,10^. Simultaneously, myofibroblasts activate TGF-β, and Wnt/β-catenin signaling to escape apoptosis and improve survival^13^. However, reactive fibrosis and cardiac remodeling lead to cardiac dysfunction^9,14^.

Notably, cell signaling pathways have critical roles in regulating these pathophysiological conditions. Some cell signaling pathways such as Notch, nuclear factor erythroid-derived 2-related factor 2/heme oxygenase-1 (Nrf2/HO-1), Ras homolog family member A/Rho-associated coiled-coil containing protein kinase (RhoA/ROCK), as well as Sonic hedgehog pathways regulate cardiac regeneration, reactive fibrosis, and cardiac hypertrophy, mediate the survival, proliferation, apoptosis, differentiation and other phenotypes of cells^12,15–19^. In general, considering cell signaling pathways as a regulating network that participate in a variety of processes after MI, it is pivotal to comprehend the mechanism of pathophysiological processes post MI. And understanding the signal transduction of molecular events eventually contributes to the recognization of the influence of signaling pathways on the progress of MI, and further leads to the discovery of novel therapeutic strategies.

Over the past few decades, enthusiastic attempts have been made to improve post-infarction prognosis in MI by targeting signaling pathways, which are known as emerging therapies, including pharmacotherapy, gene therapy, protein therapy, cell therapy, and exosome therapy^12,20,21^. These therapies address the essential causes of MI progression by targeting key signaling pathways. For example, inhibition of the Toll-like receptor 4 (TLR4)/MyD88/nuclear factor-κB (NF-κB) and TGF-β pathways alleviate excessive inflammation and cardiac fibrosis^22,23^. On the other hand, enhancing activation of the PI3K/Akt and mitogen-activated protein kinase (MAPK) pathways promotes the formation of functional vasculatures^24^. Apart from the anti-fibrosis strategy, the anti-inflammation, and therapeutic angiogenesis strategies targeting molecular mechanisms have also been well confirmed and applied for the treatment of MI^9,11,15,25,26^. Over the past decade, more advanced studies have shown that promoting the proliferation of pre-existing cardiomyocytes to drive endogenous cardiac regeneration by regulating Hippo/Yes-associated protein (YAP) signaling is viable, as another means of treating cardiac ischemic injury^27–29^.

To date, increasing numbers of preclinical studies and clinical trials were designed to pursue effective therapeutic strategies for MI. From this perspective, comprehending and summarizing the existing evidence of cell signaling pathways associated with the development and treatment of MI are essential and promising. Therefore, in this review, we explore the roles of several key signaling pathways in MI: PI3K/Akt, Notch, TGF-β/SMADs, Wnt/β-catenin, NLRP3/caspase-1, TLR4/MyD88/NF-κB, Nrf2/HO-1, RhoA/ROCK, MAPK, JAK/STAT, Hippo/YAP, and Sonic hedgehog pathways. Herein, we discuss the crucial functions of these signaling pathways in pathophysiological conditions post ischemia, all of which are promising therapeutic targets in the therapeutic strategies of MI.

## PI3K/Akt pathway in MI

**Fig. 2:**
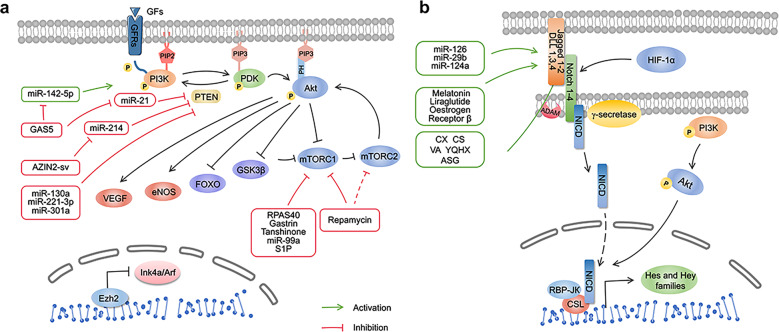
**a** PI3K/Akt signaling pathway and targeted therapy in Myocardial infarction (MI). PI3K/Akt is involved in the regulation of cardiac remodeling, regeneration, and repair post-ischemia. This pathway responds to the stimulus, likewise growth factor/growth factor receptor signaling and so on. Phosphorylated PI3K and Akt activate the downstream molecules, VEGF, eNOS, while inhibiting mTOR(C1), GSK-3β, FOXO, respectively. GF, growth factor; GFR growth factor receptor, PI3K Phosphoinositide-3 kinase, Akt protein kinase B, PIP2 phosphatidylinositol 4,5-bisphosphate, PIP3 phosphatidylinositol 3,4,5-trisphosphate, PDK phosphoinositide dependent kinase, PH Pleckstrin homology, PTEN phosphatase and tensin homolog, VEGF vascular endothelial growth factor, eNOS endothelial nitric oxide synthase, mTORC1/2 mammalian target of rapamycin complex 1/2, GSK-3β glycogen synthase kinase 3β, FOXO forkhead box subfamily O, AZIN2-sv lncRNA-AZIN2 splice variant, S1P sphingosine-1-phosphate, Ezh2 enhancer of zeste homolog 2. **b** Notch signaling pathway and targeted therapy in MI. RBP-JК recombination signal-binding protein-JК, NICD notch intracellular domain, CSL CBF1/Rbpj (mammalian), Su(H) (Drosophila), and Lag-1 (*Caenorhabditis elegans*), CX Chuanxiong, CS Chishao VA velvet antler, YQHX Yiqihuoxue prescription, AGS astragaloside

The PI3K/Akt pathway has been identified as a key mechanism in the occurrence, progression, and treatment of MI^30^. An increasing number of studies have found that the components of this pathway are activated in response to cell-external or -internal stimuli^31,32^, implicated in survival, proliferation, apoptosis, migration, and other physiological or pathological processes^30,33–35^. When PI3K converts phosphatidylinositol 4,5-bisphosphate (PIP2) into phosphatidylinositol 3,4,5-trisphosphate (PIP3), Akt is activated as the core molecule in the pathway^36,37^. PIP3 binds to the Pleckstrin homology (PH) domain of Akt to alter its conformation, exposing Ser473 and Thr308 sites^36^. Finally, phosphoinositide dependent kinase 1 (PDK1) and PDK2 phosphorylate Thr308 and Ser473 of Akt, regulate cardiac recovery following MI via the downstream signaling pathway^36,38^ (Fig. 2a).

### Downstream molecules of the PI3K/Akt pathway in MI

As downstream effectors of Akt, endothelial nitric oxide synthase (eNOS)^39^, vascular endothelial growth factor (VEGF)^40^, mammalian target of rapamycin (mTOR)^33^, glycogen synthase kinase 3β (GSK-3β)^41^, and forkhead box subfamily O (FOXO)^42^ govern cell growth, proliferation, apoptosis, and cardiovascular homeostasis (Fig. 2a).

eNOS is a member of the family of NOS enzymes encoded by *Nos2*, that catalyzes the conversion of l-arginine into nitric oxide (NO). In the heart, *Nos2* is expressed in vascular endothelial and smooth muscle cells, cardiomyocytes, and cardiac fibroblasts. NO has been proven to be a key mediator in cardiac remodeling^39^. Deletion of eNOS induced the profibrotic effect, resulting in excessive cardiac fibrosis^39^, which might provide a therapeutic target for myocardial fibrosis through activation of eNOS. In addition, activation of eNOS contributes to myocardial angiogenesis^43^, similar to the role of VEGF in therapeutic angiogenesis post MI.

Studies have shown that mTOR consists of two complexes, mTOR complex 1 (mTORC1) and mTORC2. They are both essential for cardiac remodeling following MI, because they regulate apoptosis, autophagy^44–46^, and inflammation^47^. Upregulation of autophagy is a cardioprotection mechanism response in stress^48,49^. Autophagy can be inhibited by the activity of mTORC1^50^, leading to reduced survival of cardiomyocytes in an in vitro injury model and aggravating infarction in vivo in myocardial ischemia^51^. Nevertheless, mTORC2 primarily responds to stimulation of insulin and insulin-like growth factors, which seem to also regulate cell proliferation and polarity^52–54^, protecting the heart from ischemic damage^45^. Furthermore, GSK-3β alleviates the inhibition of autophagy mediated by mTORC1 in myocardial cells and aggravates ischemic injury after prolonged myocardial ischemia^55^.

FOXOs are not only involved in tumorigenesis but are also involved in the deterioration of MI, in particular, FOXO3^56,57^. It has been noted that, following ischemia, constitutively active FOXO3a is associated with poor prognosis, resulting in deficient angiogenesis due to the increase in apoptosis and a reduction in proliferation in vascular smooth muscle cells (VSMCs)^42^. The signaling stimuli of growth factors phosphorylate Akt1 and FOXO3a, limit FOXO3a transcriptional activity, and enhance cardiomyocyte survival and native angiogenesis in the aftermath of an ischemic event^35,58^.

### The PI3K/Akt pathway as a beneficial signaling mechanism for MI therapy

#### Drugs

Phosphatase and tensin homolog (PTEN) is widely considered to be a negative regulator of PI3K/Akt by dephosphorylating PIP3 to PIP2^59,60^, participating in pathological processes in ischemic myocardium^61,62^. In preclinical studies, pharmacological inhibitors of PTEN, including HOpic^61^ and VO-OHpic^63^, have shown admirable efficacy in reducing the inhibition of PI3K and promoting angiogenesis^61^, apoptosis resistance, and survival^63^. Moreover, emerging evidence confirmed that PTEN is involved in cardiac remodeling post infarction, the decrease of PTEN activity was associated with subsequent reductions in leukocyte infiltration, cardiomyocyte proliferation, and adverse cardiac remodeling^62,64^.

As mentioned above, mTOR-dependent signal transduction is implicated in cardiac remodeling, and an mTOR inhibitor has been verified to augment autophagy and limit the infarct size of ischemia myocardium^44,65^. Rapamycin and its derivatives are common therapeutic agents that reinforce autophagy but also limit apoptosis^33,66–68^. Moreover, sphingosine-1-phosphate and tanshinone IIA have been highlighted as potential therapeutic targets that inhibit mTOR to promote angiogenesis and encourage myocyte autophagy following MI^69,70^.

#### Protein therapy and Gene therapy

With the application of recombinant proteins and viral vectors in cardiovascular diseases, increasing studies are attempting to use developing techniques for cardiovascular disease treatment^71,72^. In response to gene and protein expression of FMS-like tyrosine kinase 3 upregulated by intramyocardial injection of the recombinant FMS-like tyrosine kinase 3 ligand, cardiomyocytes are protected from apoptosis, and cardiac remodeling and function of the infarct heart were improved through Akt-dependent signaling^73^. Interestingly, gene editing of SERCA2a exerted similar cardioprotective effects^74^.

Studies have shown that non-coding RNAs, including microRNAs (miRNAs), long non-coding RNAs (lncRNAs), and circular RNAs (circRNAs), represent novel therapeutic tools for MI. A growing number of studies have observed that miRNA-21^75,76^, miRNA-130a^77^, miR221-3p^78^, and miR-301^79^ are mediated by suppression of PTEN and activation of PI3K-dependent signaling. Moreover, studies on lncRNAs indicated that small nucleolar RNA host gene 1 (Snhg1) directly binds to PTEN to form a positive feedback loop with PTEN/Akt/c-Myc to induce cardiomyocyte proliferation^80^. Furthermore, miR-99a plays a cardioprotective role in postinfarction cardiac remodeling^81^.

In recent years, with the advent of the theory of competing for endogenous RNAs (ceRNAs), several studies have attempted to explore their detailed molecular regulatory mechanisms in MI^82–85^. For example, lncRNA GAS5 competes with miR-21 to inhibit the negative regulation of miR-21 to target PDCD4 and PI3K mRNAs^83^. Similarly, acts as a ceRNA to sponge miR-93-5p mediates activation of the Rac1/PI3K/Akt pathway, revealing that CircHIPK3 could be a potential target for simultaneously reducing cardiac fibrosis and apoptosis^84^. In addition, suppression the of lncRNA-AZIN2 splice variant (AZIN2-sv) to the PTEN/Akt pathway was released by absorbing miR-214-induced angiogenesis and myocardial repair^85^. LncRNA UCA1 relieves cardiomyocytes via declining miR-122 and activating the Akt/mTOR pathway^86^. Likewise, studies illustrate that lncRNA UCA1 and DANCR are cardioprotective by decreasing miRNA-mediated mTOR signaling^86,87^.

#### Cell therapy and exosome therapy

In recent decades, stem cell therapy has gained attention due to its viability and potential use in cardiac repair^21,88,89^. Stem cells secrete cytokines and extracellular vesicles to modulate the processes following MI^21,76,90^. Transplanted bone-marrow endothelial progenitor cells (EPCs) in the myocardium trigger PI3K/Akt/FoxO signaling underlying the existence of Period 2^91^. Another study mentioned that bone marrow-derived mesenchymal stem cells (BMMSCs) release paracrine factors that exert a protective effect on cardiomyocytes against hypoxia based on overexpression of *Akt1*^92^. However, due to the unfavorable survival rate of regenerative cells, it is necessary to explore novel strategies to improve the efficacy of stem cell therapy^21^. Improving stem cell engraftment and reparative potency in injured cardiac tissue might be an alternative. Human-induced pluripotent stem cell-derived cardiomyocytes (hiPSC-CMs) and thymosin β4 microspheres were simultaneously injected into pigs after MI induction, and the microspheres delivered thymosin β4 to improve the engraftment and reparative properties of stem cells post-transplantation by heightening Akt activity^93^. In addition, relying on activation of the Akt pathway, nerve growth factor nanoparticles enhanced the therapeutic potency of human umbilical cord mesenchymal stem cells (hUCMSCs)^94^ and paracrine effects on Akt-modified BMMSC-mediated cardiac protection and functional improvement^92,95^, similar to the cardioprotective effects of edaravone-treated^96^, EGb761-treated^97^ TMSB4-transfected^98^ or IP6K-inhibited^99^ BMMSCs and rosuvastatin-supplemented adipose-derived stem cells (ADSCs)^100^.

As a possible modality that may supplant cell therapy, exosome therapy is an emerging novel approach for the treatment of MI^76,90,101^. Based on the evidence of in vivo experiments and exosomal miRNA arrays derived from human explant-derived cardiac stem cells (CSCs), exosomes from healthy donors exhibited a scarcity of heart protection compared to exosomes from patients with heart failure, and exhibited an impaired ability by blunting miR-21-5p/PTEN/AKT^102^. In addition, exosomes secreted from aged mesenchymal stem cells (MSCs) enhanced the angiogenesis and survival of cardiomyocytes via the miR-221-3p/PTEN/Akt pathway^78^. By switching PI3K signaling, analogously, exosomes excreted from SDF1-overexpressing MSCs displayed an advantageous effect on myocardial cells and cardiac endothelial cells after ischemia^103^.

## Notch signaling pathway in MI

The Notch signaling pathway has been demonstrated to play a critical role in mammalian cardiac development. During embryonic heart development, Notch1 is highly expressed in immature myocardium and expressed at low levels in postnatal myocardium. Notch1, Hes1 and Jagged1 levels in adult hearts are very low at birth. However, their levels in cardiomyocytes are significantly increased 4 days after MI^104^, suggesting that the Notch signaling pathway is involved in the regulation of myocardial injury. Many studies have found that Notch signaling induces stem cell differentiation^105^, promotes neovascularization^106^, and alleviates myocardial fibrosis^107^ and other multiple effects^108^, further mediating the repair of myocardial ischemic injury and improving cardiac function^109^. Other studies have also shown that activation of Notch signaling limits the range of myocardial ischemia and improves myocardial function after MI^110^. Additionally, there is evidence indicating that the Notch pathway is associated with the improvement of MI by improving angiogenesis^108,111,112^, improving cardiac regeneration and cardioprotection^108,113^, and reducing fibrosis^107^, apoptosis^114^, and oxidative stress^108,115^.

### The Notch pathway improves angiogenesis

Notch signaling also has physiological effects on the phenotype and functional differentiation of vascular endothelial cells. Notch1, Notch4, Jagged1, DLL-1, and DLL-4 are all expressed in endothelial cells, and only the correct binding of ligands and receptors can induce normal endothelial cell function^116^ (Fig. 1b). Notch1 acts as a mechanical sensor in adult arteries, where endothelial cells transform mechanical forces into intracellular signals^116^. Intracellular signals are essential for vascular homeostasis, junction integrity, and endothelial cell elongation^116^. The Notch pathway is also correlated with VEGFA signaling in regulating the differentiation of endothelial cells, the sprouting of capillary networks, and the branching and fusion of endothelial tubes^117^.

### The Notch pathway reduces myocardial fibrosis

Cardiac fibroblasts proliferate and differentiate into myofibroblasts after myocardial injury, express smooth muscle actin (SMA), secrete collagen, and participate in tissue repair^108^. However, progressive fibroblast proliferation and differentiation result in the excessive synthetic secretion of collagen, eventually leading to myocardial fibrosis^108^.

The Notch pathway plays a crucial role in myocardial fibrosis. It directly regulates the expression of α-SMA through activation of the primary effector CSL in endothelial cells and vascular smooth muscle cells^118^. Many studies have suggested that activation of the Notch1 signaling pathway prevents myocardial fibrosis. For example, Notch1 knockout mice were more likely to develop myocardial fibrosis after myocardial injury than wild type mice^119^, while enhanced Notch1 activity inhibited the transformation of fibroblasts into myoblast fibroblasts by antagonizing TGF-β1/SMAD3 signaling^107^. Moreover, some therapies have been developed to explore the application of stem cells or miRNAs to decrease fibrosis^110,120^. For example, investigators transplanted N1ICD-overexpressing C-MSCs into MI mice and observed decreased myocardial fibrosis after MI^110^. Another study also used miR-29b to inhibit myocardial fibrosis by activating the Dll4-Notch1-Hes l signaling pathway in MI rats^120^.

Besides Notch1, Notch3 reportedly inhibits cardiac fibroblast proliferation, promotes apoptosis, and reduces the transition of fibroblasts to myofibroblasts^121^. They found that Notch3-mediated cardiac fibroblast activity by negatively regulating the RhoA/ROCK/HIF-1α-signaling pathway^121^. In addition, expression of Notch-4 was also observed in cardiac fibroblasts^118^.

### The Notch pathway reduces cardiomyocyte apoptosis

In vitro and in vivo studies have suggested that the Notch pathway plays a significant role in reducing cardiomyocyte apoptosis^114^. In an in vitro experiment in a hypoxic cardiomyocyte model, Notch1-regulated apoptosis by down-regulating Bcl-2 and Bax and up-regulating caspase-9 and -3^114^. At the same time, the Notch signaling pathway exerts an anti-apoptotic effect by regulating the transcription factor RBP-J in MI mice^122^. Additionally, another study reported that Notch1 inhibits the binding of NF-КB to DNA, thereby playing a negative regulatory role in inhibiting apoptosis and enhancing cell survival^123,124^.

### The Notch pathway reduces oxidative stress in cardiomyocytes

The function of the Notch pathway in antioxidative stress has been reported in several studies^105,125,126^. For instance, TNF-α inhibitor was demonstrated to suppress oxidative stress in myocardial ischemia/reperfusion (I/R) injury partly through Notch1 signaling^125^. Considering that the Notch pathway correlates with antioxidative stress, researchers have developed several therapeutic methods and stem cells to upregulate Notch1 signaling to reduce oxidative stress^105,126^. Overexpression of aldolase A (ALDOA) decreases the hypoxia/reperfusion-triggered oxidative stress and apoptosis in cardiomyocytes by upregulating VEGF/Notch1/Jagged 1 axis^126^. Another study used EV-C-MSCs carrying N1ICD and found that they decreased the apoptosis of endothelial cells and cardiomyocytes under oxidative stress and ischemic injury in vitro^105^.

### The Notch pathway in the improvement of cardiac regeneration and cardioprotection

During the early postnatal stage, Notch pathway activation is important for regulating cardiomyocyte proliferation^127^. Notch signaling plays a crucial role in cardiac development, guiding cell fate decisions that underlie myocyte, and vessel differentiation^127^. In adults, Notch signaling is inhibited in healthy individuals because epigenetic modification of the Notch pathway suppresses cardiac regeneration ability^127^. However, Notch signaling is activated when injury, hypoxia, and diseases are encountered.

It was reported that reactivation of the Notch pathway is crucial for adult zebrafish to drive cardiac regeneration after injury and in HMGB1-mediated cardiac regeneration^128,129^. In addition, it also promotes the growth, survival, and differentiation of cardiac progenitor cells into smooth muscle lineages in vitro^130^. Another study knocked out the Notch1 gene in bone marrow-derived stem cells to treat MI mice, and they observed impaired cardiac repair, suggesting that the Notch signaling pathway plays an important role in the myocardial repair of bone marrow-derived stem cells^131^.

Besides the role of the Notch pathway in cardiac repair, much preclinical and clinical evidence has also revealed the cardioprotective role of Notch signaling pathways. In a high glucose cell model of hypoxic injury, the Jagged1-Notch signaling pathway exerts a cardioprotective effect^113^. Another study suggested that upregulation of Notch3 and Notch4 mRNA levels, as well as NICD-3 and -4 in cardiomyocytes induces therapeutic benefits in chronic HF^132^. Furthermore, clinical evidence is also emerging for the use of Notch1 signaling-activated BMMSCs in patients with ischemic heart disease^131^.

### Correlation between Notch and other signaling pathways in MI

#### Akt signaling

Notch signaling is reportedly activated by the C-Met/HGF and PI3K/Akt signaling pathways after myocardial injury. Interestingly, Notch also enhances the expression of PI3K/Akt signaling in adult myocardium following myocardial injury^110^. This mutually supportive crosstalk suggests a positive survival feedback mechanism between Notch and Akt signaling^110^.

#### Notch signaling and hypoxia

The imbalance between oxygen supply and oxygen consumption during hypoxia activates oxygen transport and hypoxic cellular metabolism pathways^133^. Studies have confirmed that Notch signaling is sensitive to hypoxia, and there are multiple direct and indirect interactions between Notch signaling molecules and the hypoxia-inducible factor (HIF) signaling pathway^133^. First, hypoxia activates the Notch pathway. The gradual accumulation of HIF in tissues stimulates the Notch signaling pathway by activating the expression and synthesis of the exogenous intracellular domain (NICD) promoter to initiate expression of the downstream genes Hes1 and Hey2^134^. Moreover, inhibition of miR-363 protects cardiomyocytes against hypoxia-induced apoptosis through the promotion of Notch1 expression and the activation of Notch signaling^135^.

Second, the Notch pathway and hypoxia exert synergistic effects. For example, myocardial ischemia also activates the Notch signaling pathway and induces HIF expression by expressing the target gene Hesl^136^, alleviating myocardial I/R injury^136^. Moreover, the HIF-1α-Notch1 pathway is required for the generation of arterial endothelial cells for arteriogenesis and revascularization of ischemic tissue^134^. This synergistic effect of HIF-1α and the Notch signaling pathway maximizes the rescue of damaged myocardia.

Hypoxia induces expression of Notch ligand Dll 4 and target genes Hey1 and Hey2, activating the Dll 4-Notch-Hey 2 signaling pathway, whose activation is dependent on the activation of HIF-1α and Notch^137^. Elevated expression of Dll 4 and Hey2 in endothelial progenitor cells inhibits the chicken ovalbumin upstream promoter transcription factor II (Coup-TF II), regulating the production of arteries^138^. Hey inhibits HIF-1α-induced gene expression^136^, which suggests that there is negative feedback to prevent hypoxia-induced gene overexpression^139^.

### Application of the Notch pathway in intervention therapy for MI

To date, there is very limited evidence regarding the application of Notch in clinical therapy. Previous studies investigated whether the Notch signaling-induced proangiogenic effect may be the reason for the beneficial effect after the treatment of MI using traditional Chinese medicine and cell therapy^62,133,140–145^. Many studies have reported that the regulation of non-coding RNAs including miRNAs^123,146–154^, lncRNAs^155,156^, and circRNAs^147^ could exert a therapeutic role in myocardial repair. Moreover, some drugs are reported to correlate with the Notch pathway^157–161^. For example, it has been reported that Notch signaling participates in the antiapoptotic effects of liraglutide on cardiomyocytes against high glucose-induced myocardial damage^157^. Oestrogen receptor β activation enhances Notch1 signaling and its downstream mediator-PI3K/Akt signaling to improve myocardial function in MI model^158^. Although previous studies have suggested that the Notch signaling pathway may be a target of treatment for MI, most are preclinical evidence. Therefore, it is of great significance to further explore the role of Notch signaling in all possible therapies in clinical practice. Up to now, the benefit of melatonin, a regulator of Notch1/Mfn2 pathway^145^, has been investigated in many clinical trials for coronary heart disease and shows a potential promising clinical application value in reducing infarction size^162^. However, some evidence suggested melatonin did not improve the myocardial salvage^163^. It remains to be studied whether melatonin protects the adverse myocardial remodeling in patients with MI.

## NLRP3/caspase-1/IL-1β signaling pathway in MI

**Fig. 3:**
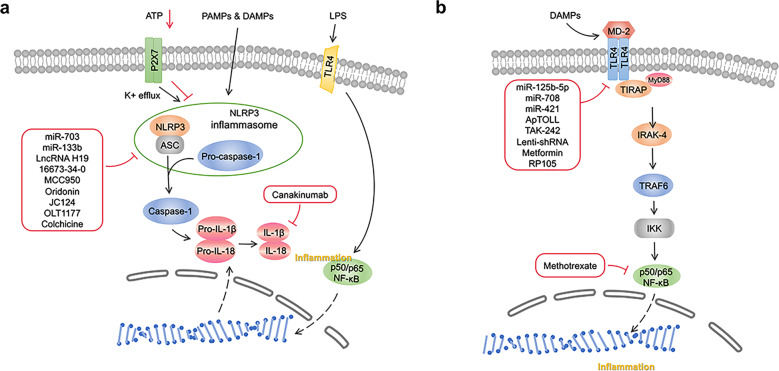
**a** NLRP3/caspase-1 signaling pathway and its correlated intervention after MI. NLRP3/caspase-1 inflammasome pathway mediated inflammation, pyroptosis, oxidative stress, fibrosis, cardiac remodeling following MI. When NLRP3 is activated by DAMPs and PAMPs, it binds to ASC adaptor molecule and aggregates with pro-caspase-1. Then the NLRP3 inflammasome converts pro-caspase-1 to caspase-1, which catalyzes the conversion of pro-IL-1β and pro-IL-18 to its mature product IL-1β and IL-18. ATP adenosine triphosphate, LPS lipopolysaccharide, PAMPs pathogen-associated molecular patterns, DAMPs Danger-associated molecular patterns, TLR4 toll-like receptor 4, NLRP3 nucleotide-binding domain, leucine-rich-repeat family, pyrin-domain-containing 3, ASC activating signal cointegrator, IL interleukin, NF-κB nuclear factor-κBn, OLT1177 Dapansutrile. **b** TLR4/MyD88/NF-κB signaling pathway and its correlated intervention after MI. TLR4/MyD88/NF-κB signaling pathway mediated inflammation, pyroptosis, apoptosis, fibrosis, ventricular arrhythmias and lipid metabolism after myocardial infarction. Cardiac injury generates endogenous signals that activate the TLR4/MyD88/NF-κB signaling pathway. The activation of the TLR signaling pathway originates from the cytoplasmic TIR domain that associates with a TIR domain-containing adaptor, MyD88. This signaling pathway activates NF-κB, a transcription factor, and subsequently induce the production of proinflammatory cytokines. TLR4 Toll-like receptor 4, TIRAP TIR (Toll/IL-1 receptor) domain-containing adapter protein, IRAK-4 IL-1 receptor-associated kinase-4, TRAF6 tumor necrosis factor receptor-associated factor 6, IKK IκB kinase, NF-κB Nuclear factor-κB, Lenti shRNA Lentivirus short hairpin RNA, TAK-242 resatorvid, RP-105 radioprotective 105

Some studies suggest that imbalanced inflammation facilitates adverse myocardial remodeling through the activation of one of the most well-known innate inflammatory signaling pathways, the nucleotide-binding domain, leucine-rich-repeat family, pyrin-domain-containing 3 (NLRP3)/caspase-1 inflammasome pathway^164,165^. It has also been shown that the NLRP3 inflammasome plays an indispensable role in the development and progression of inflammation in MI^166^ (Fig. 3a).

### Activation of the NLRP3/caspase-1 inflammasome pathway in MI

The canonical NLRP3 inflammasome is an intracellular protein complex consisting of the NOD-like receptor (NLR) family member NLRP3, the adaptor protein apoptosis-associated speck-like protein containing a caspase-activating and recruitment domain (ASC), and pro-caspase-1^167^. PRRs, such as Toll-like receptor 4, recognize a priming signal of infection or tissue damage to activate the inflammatory transcription factor NF-kB, which increases NLRP3, pro-interleukin (IL) -1β, and pro-IL-18^168^. When NLRP3 is activated, it binds to the activating signal cointegrator (ASC) adaptor molecule and aggregates with pro-caspase-1. Then, the NLRP3 inflammasome converts pro-caspase-1 to caspase-1, which catalyzes the conversion of pro-IL-1β and pro-IL-18 to its mature products IL-1β and IL-18^169^. IL-1β and IL-18 cause inflammation and tissue damage by regulating immune cell recruitment, cytokine production, and extracellular matrix turnover in the inflammatory response following MI^170,171^ (Fig. 3a).

Increasing evidence shows that MI is accompanied by inflammatory responses that lead to leukocyte accumulation, the release of inflammatory cytokines/chemokines, myocardial damage, healing, and scar formation^172^. Therefore, it is important to preserve the heart function and prevent the development of adverse remodeling through timely repression and containment of inflammatory signals^173^. Several studies focusing on the relationship between NLRP3 inflammasome activation and patients with MI have been reported. Defects in the inflammasome and associated proteins may be involved in promoting ischemic heart disease^174^.

### The NLRP3/caspase-1 inflammasome pathway-mediated inflammation, pyroptosis, oxidative stress, fibrosis, and cardiac remodeling following MI

Many molecules and transcription factors participate in the regulation of the NLRP3/caspase-1 inflammasome pathway in MI. Several studies have shown that nicorandil, isofraxidin, resveratrol (RES, a naturally occurring polyphenol), and short-term aminooxyacetic acid (an inhibitor of aspartate aminotransferase in the aspartate-arginosuccinate shunt) exert cardioprotective effects through inhibition of the NLRP3 inflammasome to reduce MI-induced inflammation^175–177^. Meanwhile, the inhibition of glycogen synthase kinase-3β or cathepsin B also alleviates activation of the NLRP3 inflammasome in MI^178,179^. Furthermore, several factors, such as nicorandil^180^ and growth differentiation factor 11^181^, exert cardioprotective effects by inhibiting the NLRP3/caspase-1 inflammasome pathway to reduce MI-induced pyroptosis. A recent study investigated whether the NLRP3/caspase-1 pathway also plays a unique role in regulating oxidative stress^182^. In addition, salvianolate and resveratrol reduce cardiac fibrosis by inhibiting NLRP3 inflammasome signaling and the TGF-β1/SMAD2 signaling pathway in post-MI rats^176,183^. Moreover, NLRP3 inflammasome activation plays an essential role in cardiac remodeling and malignant ventricular arrhythmia after MI^165,179,184–186^. Besides the cardiac cells, deficiency of the epigenetic regulator Tet2 in hematopoietic cells is associated with elevated IL-1β-NLRP3 inflammasomes to induce greater cardiac dysfunction^185^. In addition, a previous study focused on the deterioration of bone vascular function in ischemic heart disease and found that inhibition of NLRP3 partially prevented the loss of type H vasculature after MI in mice^187^.

Some non-coding RNAs also regulate NLRP3/caspase-1 levels in MI. Recent studies have shown that miR-703^188^ and miR-133b^189^ attenuate pyroptosis and hypoxia injury by inhibiting NLRP3/caspase-1 after MI. Moreover, in hypoxic cardiomyocytes, lncRNA H19 overexpression also inhibits NLRP3/caspase-1 to suppress the cell apoptosis rate and promote the cell proliferation rate^190^.

Furthermore, MSCs exosome treatment reduces white blood cell accumulation and expression of the NLRP3 inflammasome around the infarct area in mouse hearts subjected to left coronary artery (LCA) ligation^191^. Increased NLRP3 inflammasome activity also plays a role in the pathogenesis of aging-related functional decline in human ADSCs in the aging hosts^192^. As such, the NLRP3 inflammasome is a key mediator of the post-MI inflammatory response and tissue injury.

### Clinical prospects of the NLRP3/caspase-1 inflammasome pathway

As mentioned above, preclinical studies have shown that inhibition of the NLRP3 inflammasome has beneficial effects on preventing infarction injury after MI. Hence, many inhibitors have been developed based on the functional effect of this molecule regarding the treatment of MI. Pharmacological inhibition of the NLRP3 inflammasome via an NLRP3 inflammasome inhibitor (16673-34-0), an intermediate in the synthesis of glyburide, limits cell death and left ventricle systolic dysfunction after ischemia in mice^193^. Porcine MI models treated with the NLRP3-inflammasome inhibitor MCC950 (6 or 3 mg/kg) markedly preserve the left ventricular ejection fraction^194^. Moreover, Li, X., et al. noninvasively demonstrated the therapeutic effects of MCC950 in AMI using (18)F-FDG PET imaging^195^. The covalent NLRP3 inflammasome inhibitor oridonin reduces expression levels of NLRP3, IL-1β, IL-18, and myocardial fibrosis and preserves cardiac function in a mouse MI model^196^. JC124, a benzenesulfonamide analog used as an NLRP3 inflammasome inhibitor, is now being further studied in mouse models of acute MI, but the results have not yet been published^197^. OLT1177 (dapansutrile), a β-sulfonyl nitrile molecule and a novel NLRP3 inflammasome inhibitor, preserves myocardial function in I/R or non-reperfused anterior MI mouse models^198,199^.

Previous studies found that increase of ATP levels following ischemia/reperfusion stimulates P2X7-mediated release of IL-1β, IL-18, and ROS, promoting myocardial damage and declining cardiac function^200,201^. In contrast, inhibition of P2X7 (brilliant blue G) abrogates the protective ATP-driven effect of short bouts of I/R conditioning and results in increased infarct sizes^202^. Additionally, colchicine (a drug with broad anti-inflammatory effects, including inhibitory effects on the NLRP3 inflammasome)^203^ and canakinumab (inhibition of IL-1β)^204^ have shown efficacy in preventing major adverse cardiovascular events in phase III trials in patients with ischemic heart disease.

There are also several large, randomized placebo-controlled trials. For example, CANTOS^205^ tested subcutaneous canakinumab 300 mg every 3 months against placebo in patients with a history of MI and serum C-reactive protein (CRP) > 2 mg/L, demonstrating efficacy in preventing major cardiovascular events but increased rates of fatal infections. COLCOT^206^ (in patients with recent MI) and LoDoCo2^207^ (in patients with chronic coronary syndromes) tested oral colchicine 0.5 mg daily vs. placebo, demonstrating prevention of major cardiovascular events with a slightly increased risk of pneumonia in COLCOT (0.9% vs. 0.4%) but not in LoDoCo2. Expanding translational research using selective NLRP3 inhibitors is necessary to fully evaluate the potential of NLRP3 inflammasome inhibition in cardiovascular disease.

## TLR4/MyD88/NF-κB-signaling pathway in MI

Innate immune cells identify danger signals via engagement of Toll-like receptors (TLRs), a family of transmembrane receptors that activates downstream pro-inflammatory cascades^208^. TLRs are an important class of protein molecules involved in non-specific immunity that serve as a bridge between non-specific and specific immunity, as well as recognizes invasion and activates the immune response^209^. To date, more than 10 TLRs have been identified. TLR4 has been the most studied TLR and is widely present on the surface of a variety of cells, such as macrophages^210^, dendritic cells^211^, endothelial cells^212^, and epithelial cells^213^.

Functional enrichment analyses of 134 genes (gene expression omnibus, GEO database) from patients with different phases of MI identified several hub genes (IL1R1, TLR2, and TLR4) associated with the progression of MI, which can be used as new diagnostic molecules for MI^214^. Previous cardiac studies have shown that the activation of TLR4 causes increased expression of proinflammatory cytokines, leading to inflammatory responses and additional damage to the already injured myocardium^172^. Notably, the TLR4-signaling pathways correlate with infarct severity but not with the extent of inflammation. TLR4 and downstream gene expression profiles are upregulated in both infarcted and remote myocardium following MI^215,216^. In addition, necrotic cardiac myocytes release a wide range of endogenous signals due to MI (S100A1, S100A8/A9, HMGB1, galectin-3, S100β, IL-1α, etc.), associated with significant TLR4 induction^217–219^. Moreover, platelet activating factor receptor (PTAFR), TLR4, miR-149-5p, miR-6778-3p, and miR-520a-3p were found to be involved in the progression of stable coronary artery disease to AMI in a clinical study^220^. Conversely, a recent study showed that patients with ST-segment elevation MI have increased expression of a series of genes that implicate NF-κB activity, including HIF-1α, NF-κBIα, IL-18R1/2, MMP9, and IL-8, but reduced expression of TLR4-induced genes, such as TNF-α^221^. Therefore, further studies focused on the expression of TLR4 and downstream genes in different stages and categories of cardiac disease are needed to confirm these findings (Fig. 2b).

### The TLR4/MyD88/NF-κB-signaling pathway mediates inflammation, pyroptosis, apoptosis, fibrosis, ventricular arrhythmias and lipid metabolism after myocardial infarction

Some molecules or transcription factors participate in the regulation of TLR4/MyD88/NF-κB in MI. Gentianella acuta, astaxanthin, astragaloside IV, and danshen (*Salvia miltiorrhiza*) may ameliorate inflammatory injury via the TLR4/MyD88/NF-κB signaling pathway after acute MI^222–225^. On the other hand, Li et al. indicated the involvement of the TLR4/MyD88/NF-κB/NLRP3 signaling pathway in attenuating pyroptosis in MI rats treated with nicorandil^180^. Inhibition of the TLR4/TNF-α signaling pathway in dapsone-mediated cardioprotection also ameliorates apoptosis in rats^226^. Moreover, the TLR4/MyD88/NF-κB pathway plays a unique role in ameliorating myocardial fibrosis via modified citrus pectin^23^. Activation of the TLR4/CaMKII signaling pathway is related to vulnerability to ventricular arrhythmias in myeloid differentiation protein 1 (MD1) deletion mice after MI^227^.

In addition, some metabolism-related factors are also involved in the regulation of the TLR4/MyD88/NF-κB pathway as follows: HIF-1α and apolipoprotein A-I mimetic peptide 4F (4F) may attenuate myocardial injury by minimizing TLR4 upregulation in post-MI rats^228,229^; cardiac TLR4 is preferentially upregulated by oxidized cholesterol in rats with MI^230^. Similarly, activation of the TLR4-MyD88 signaling pathway in a hyperlipidemic environment inhibits the lisinopril-mediated cardioprotective effect^231^. Moreover, electroacupuncture, a physiotherapy factor, may alleviate the excessive inflammatory response after MI by inhibiting the expression of the IL-23/IL-17 axis in MI rats, and TLR4 may be involved during the process^232^. As such, targeting these factors during different phases of MI may offer an effective therapeutic approach for preserving the function of the ischemic heart.

Some non-coding RNAs are also involved in regulating the TLR4/MyD88/NF-κB signaling pathway in MI. Previous studies have shown that miR-125b-5p, miR-708, and miR-421 attenuate anoxia/reoxygenation injury and the inflammatory response by blocking TLR4 signaling via targeting circRNA nuclear factor IX^233^, HMGB1^234^, and JAK2/STAT3^235^. Furthermore, M1 macrophage-derived extracellular vesicles may promote cardiac dysfunction through TRL4-dependent NF-κB^236^. Moreover, MSCs exosomes attenuate myocardial ischemia injury in mice by shuttling miR-182/TLR4, which modifies the polarization status of macrophages^237^. These studies shed new light on potential therapeutic tools for myocardial ischemic injury.

### The clinical perspective of TLR4/MyD88/NF-κB inhibition

Sustaining TLR4 activation may lead to deleterious myocardial inflammation; hence, studies have explored several approaches regarding the negative regulation of TLR4. Many preclinical studies focused on inhibiting the TLR4/MyD88/NF-κB signaling pathway have shown beneficial effects in preventing infarction injury after MI. The TLR4 antagonist, ApTOLL^238^ may be effective in an in vivo pig model of AMI by decreasing inflammatory production of IL-1β and IL-6 and increasing production of IL-10. In addition, radioprotective 105 (RP105), a TLR4 homolog that competitively inhibits TLR4 signaling, confers protective effects on cardiac function after MI^239^. Moreover, the nanoparticle-mediated administration of TAK-242, a chemical inhibitor of TLR4, attenuates AMI injury by regulating TLR4-dependent monocyte/macrophage-mediated inflammation in a mouse model^240^. In addition, the clinical drugs metformin and methotrexate, act as TLR4 and NF-κB inhibitors to reduce MI size and improve cardiac function in animal post-MI models^241,242^. Furthermore, research focusing on gene therapy shows that injection of lentivirus shRNA against TLR4 into the infarcted heart significantly decreases infarct size and improves cardiac function in vivo^243^. However, the prevention or treatment of cardiac diseases using TLR4 inhibitors or antagonists has not currently been launched in human clinical trials. Further studies are still required to devise methods for protecting the myocardium from additional damage and to contribute to the treatment of MI.

## NRF2/HO-1 signaling pathway in MI

NRF2 is the product of the NFE2L2 gene and consists of seven functional domains^244^. It belongs to the Cap ‘n’ Collar (CNC) subfamily^245^. NRF2 is extremely unstable and easily degraded in a non-stress state^246^. NRF2 is an important factor that maintains ROS homeostasis and participates in the regulation of antioxidant genes^247^. It may sense oxidative signals and transfer signaling molecules to the nucleus, initiating antioxidant gene transcription^248^. In acute kidney injury, stroke, and other diseases, the use of NRF2-activated compounds effectively reduces ROS, preventing or delaying disease progression^249,250^.

Heme oxygenase (HO) is a rate-limiting enzyme that catalyzes heme to biliverdin Ixα, carbon monoxide (CO), and iron^251^. HO-1, HO-2, and HO-3 all belong to the three isoenzymes in the HO system, and all of them show the same catalytic activity^252^. As a downstream target of NRF2, HO-1 is involved in antioxidant stress and cell protection. For example, HO-1 protects retinal ganglion cells^253^, liver cells^254^, and hippocampal neurons^255^ from I/R injury. In addition, HO-1 can also enter mitochondria to regulate autophagy and inflammation in cells^256^. Therefore, the protective effect of HO-1 on myocardial cells after MI should not be ignored.

### The function of the NRF2/HO-1 signaling pathway in MI

NRF2 plays a crucial role in combating various oxidative stress responses and heart remodeling after MI (Fig. 3a). For example, in the NRF2-KO mouse model, the important role of NRF2 in protecting multiple organs, including the heart, has been widely confirmed^17,257,258^. Moreover, deletion of NRF2 induces significantly higher mortality of mice after MI is significantly higher than that of mice in the control group, demonstrating that NRF2 plays an important role in MI^17^. In addition, the important role of HO-1 in the long-term treatment and rehabilitation of MI has also been confirmed. After the modeling of acute MI in rats that received HO-1 pretreatment, in long-term follow-up observations, compared to the control group, the long-term survival rate and myocardial function are significantly increased, and left ventricle remodeling was significantly decreased^259,260^.

#### Apoptosis

NRF2/HO-1 is an important pathway that exists in almost all cells types in the body to maintain homeostasis and reduce oxidative stress^261^. The apoptosis of myocardial cells after MI is one of the important reasons leading to impaired heart function^262^. Studies have shown that wogonin^263^, hirudin^264^, dapsone^226^, and rosuvastatin combined with low-dose carvedilol^265^ all act on the NRF2/HO-1 pathway to protect cardiomyocytes from oxidative stress damage after MI and reduce cardiomyocyte apoptosis. The final outcome maintains normal cardiomyocyte function and myocardial tissue structure as well as prevents ventricular remodeling. When HO-1 is successfully activated in rabbit I/R models, it reduces the occurrence of myocardial apoptosis by inhibiting the translocation of NF-κB and AP-1^266^. In addition, pre-injection of HO-1 or HO-1 activator into the heart significantly reduced MI size and myocardial apoptosis^267,268^. All this evidence suggests that HO-1 can directly treat MI by reducing oxidative stress-induced damage.

#### Hypoxia and oxidative stress

Stem cell therapy is one of the most promising therapies in MI^269^. However, stem cells injected into the border area after MI cause a large number of deaths due to environmental effects such as hypoxia and ischemia, which reduce their therapeutic utility. Overexpression of HO-1 in stem cells effectively solves the tolerance of stem cells to hypoxia and oxidative stress, and simultaneously enhances their paracrine function, thereby increasing the survival rate and enhancing the therapeutic effects^270–272^. This provides an experimental basis for improving the therapeutic effect of stem cells in the future.

NRF2/HO-1 also protects cardiomyocytes from oxidative stress by regulating ion channels. Excessive Ca^2+^ influx leads to activation of Ca^2+^-dependent degradation enzymes, which in turn leads to cellular oxidative stress and dysfunction. Carbon monoxide is the product of HO-1 decomposing heme, which promotes the proliferation of VSMCs and protects cardiomyocytes by inhibiting L-type Ca^2+^ channels and T-type Ca^2+^ channels^273,274^. The proper function of ion channels is closely related to mitochondria. When cardiomyocytes are in an ischemic state, it leads to the deposition of excess ROS and the dysfunction of mitochondrial membrane potential^275^.

### The predictive effect of HO-1 in the blood on MI prognosis

In current clinical studies, it remains controversial whether the levels of HO-1 expression in the blood are correlated with the degree of MI. During the six-month follow-up of AMI discharge, researchers found that increased HO-1 exhibits a significant association with lower severity of coronary artery disease^276^. However, another two studies suggested the opposite conclusion. SM Chen et al. demonstrated that compared to the control group, expression levels of HO-1 in patients with stable angina pectoris, unstable angina pectoris, and acute MI displayed a rising trend related to disease severity^277^. Another cohort study of non-cardiac surgery showed that the incidence of adverse cardiac events in elderly patients with high HO-1 expression before surgery was greater than that in elderly patients with low HO-1 expression after non-cardiac surgery^278^. We think there are three possible reasons for this divergence. The first is that the source of HO-1 is the patients’ blood, and HO-1 in the blood does not fully represent the true condition of HO-1 in the damaged heart tissues. The second is that the damaged myocardium releases high levels of ROS. These increased levels of ROS do not increase the expression of HO-1^279^. Therefore, whether it is reasonable to use HO-1 in the blood to detect the level of myocardial damage needs further investigation. The last reason is that the total number of samples included was relatively small, and cannot objectively reflect the real situation of HO-1. Therefore, it is necessary to investigate HO-1 expression and MI severity in a larger population in the future.

## RhoA/ROCK signaling pathway in MI

RhoA is one of the most important members of the Rho family, and the primary function of the Rho family is widely known for its key role in regulating the cytoskeleton of actin in eukaryotic organisms. The spatiotemporal regulation of RhoA activation is responsible for cellular morphology, attachment, and cell movement^280^. Under the regulation of guanine nucleotide exchange factor (GEF), GTPase activating proteins (GAPs), and guanine nucleotide dissociation inhibitor (GDI), RhoA switches back and forth between the inactive GDP state and active GTP state to play a biological role^281^. In addition, mammalian RhoA shares a common post-translational modification region (PTM) at its carboxyl terminus (COOH)^282^. This region allows RhoA to anchor to the cell membrane, which is necessary for its activation. Only activated RhoA can bind to cell membranes and regulate signaling molecules^282^. GDI is a negative regulator of RhoA that inactivates RhoA and disconnects it from the membrane to the cytoplasm, and this effect can be reversed by GDF, which allows RhoA to anchor to the cell membrane and restart the cycle again^283^.

RhoA plays a crucial role in regulating the development and differentiation of the nervous system and cardiovascular system in the embryonic period. For example, during the development of the central nervous system, RhoA regulates neuronal migration mediated by radial glia^284^. In the cardiovascular system, the primary role of RhoA in its early formation is to promote heart tube fusion, while in the later stage of formation, RhoA plays a role in the construction of the conduction system^285,286^. In addition, RhoA also mediates the differentiation of coronary artery smooth muscle cells and epicardial cells^287^. In myocardial cells, RhoA regulates L-type Ca^2+^ currents and potassium channels^288,289^. In addition, it is a potential inhibitor of the cardiac fast Na^+^ current^290^.

ROCK is the key downstream target of RhoA. It consists of an N-terminal domain and a C-terminal cysteine-rich domain located in the PH motif domain^291^. ROCK has 2 subtypes: ROCK1 and ROCK2^292^. They contain 1354 and 1388 amino acids, respectively, and there are 65% and 55% similar homologies in their amino-acid sequence and kinase domains^293^. Therefore, they are similar in structure and function^294^. Nevertheless, due to their distinct localization of tissue and subcellular structure^295^, there are differences in their functions in certain diseases. For example, in diabetic nephropathy, ROCK1 is involved in mitochondrial dynamics and cell differentiation, while ROCK2 is related to inflammation, fibrosis, and cell death^296^. In airway hyperresponsiveness, although both ROCK1 and ROCK2 can mediate ozone-induced airway hyperresponsiveness, the mechanism is different^297^.

**Fig. 4:**
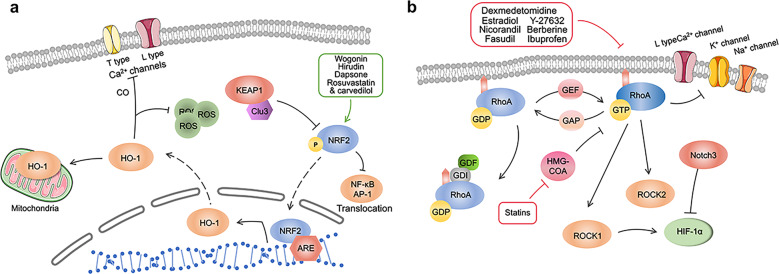
**a** Nrf2/HO-1 signaling pathway and targeted therapy post MI. NRF2/HO-1 plays a crucial role in combating various oxidative stress responses and heart remodeling after MI. It exists in almost all kinds of cells in the body to maintain homeostasis and reduce oxidative stress. In addition, this pathway also plays an important role in stem cell therapy of MI and prognosis prediction of MI. KEAP1 kelch like ECH associated protein 1, NRF2 nuclear factor erythroid-derived 2-related factor 2, HO-1 heme oxygenase-1, ARE antioxidant responsive element, ROS reactive oxygen species. **b** RhoA/ROCK signaling pathway and targeted therapy post MI. RhoA switches back and forth between inactive GDP state and active GTP state, so as to play its biological role. ROCK is a downstream molecule of Rhoa. They all play a role in fibrosis, ventricular remodeling, and cardiac repair after myocardial infarction. Many drugs, including statins, can play their role in treating myocardial infarction by targeting the RhoA/ROCK pathway. RhoA Ras homolog family member A, ROCK Rho associated coiled-coil containing protein kinase, HIF-1α hypoxia inducible factor-1α, HMG-CoA hydroxymethylglutaryl-CoA, GAP GTPase-activating protein, GDI guanine dissociation inhibitor, GDP guanosine diphosphate, GTP guanosine triphosphate, GEF guanine nucleotide exchange factor

Is there any difference between the roles of ROCK1 and ROCK2 in the heart? The answer is yes. ROCK1 cardiac-specific knockout mice exhibit myocardial hypertrophy, but cardiac-specific ROCK2 knockout mice do not display signs of myocardial hypertrophy^298–300^. These results provide evidence for further exploring the mechanism of ROCK1 and ROCK2 in cardiomyocyte hypertrophy after MI (Fig. 4b).

### The function of the RhoA/ROCK signaling pathway in MI

There is no doubt that the RhoA/ROCK signaling pathway plays a crucial role in cardiovascular diseases including MI^301^. However, the direct role of RhoA in the myocardium is rarely studied at present, but it is certain that RhoA directly or indirectly regulates the death and survival of myocardial cells, myocardial hypertrophy, and fibrosis after ischemic injury^302,18^. These effects may be related to its regulation of actin, cell morphology, and ion channel status^303^.

#### Myocardial fibrosis

Myocardial fibrosis is an important pathophysiological process in the border area after MI. It has been confirmed that HIF-1α plays an important role in fibrosis after MI^304,305^. The RhoA/ROCK signaling pathway is upstream of HIF-1α. The profibrotic effect of HIF-1α is negatively regulated by Notch3 via the RhoA/ROCK/HIF-1α signaling pathway^121^. It is of great significance to further understand the pathogenesis of cardiac fibrosis. Estradiol and nicorandil are two common clinically utilized drugs. Injecting the above two drugs into the border area of MI significantly reduced the occurrence of fibrosis by inhibiting the RhoA/ROCK signaling pathway^306,307^. Recently, fasudil, a protein kinase inhibitor based on the structure of isoquinoline sulfonamide, was approved for clinical use as the first ROCK inhibitor^308^. Although fasudil is mainly used to treat cerebrovascular diseases^309,310^, its therapeutic effect has been demonstrated in animal models with myocardial fibrosis after MI^311,312^. Its appearance offers hope for fibrosis after MI. This application prospect is worth investigating in myocardial fibrosis after MI.

#### Oxidative stress

Oxidative stress is an important pathophysiological process after MI. Numerous studies have shown that the Rho signaling pathway participates in related reactions such as oxidative stress and inflammation^313–315^. At present, ligation of the left anterior descending coronary artery and isoproterenol injection are the two primary methods for modeling MI. The former is a mechanical blockage of blood flow that leads to MI^316^. The latter causes oxidative stress in the heart, which leads to progressive mitochondrial damage and changes in cardiac biochemical parameters^317^. Therefore, the use of isoproterenol injection can be used to further explore the performance of oxidative stress after MI. At present, it has been found that dexmedetomidine^318^, berberine^319^, ibuprofen^320^, and fasudil^321^ regulate the RhoA/ROCK pathway to protect cardiomyocytes from damage caused by isoproterenol. The ultimate result of these interventions preserves heart function and prevents cardiomyocyte death and ventricular remodeling.

### Statins and MSCs in the treatment of MI by regulating the RhoA/ROCK signaling pathway

Statins are clinically important lipid-lowering drugs. Studies have shown that statins can protect the heart after MI. For example, statins such as rosuvastatin^322^ and fluvastatin^323^ protect myocardial cells and reduce apoptosis after MI by regulating the RhoA/ROCK pathway. Nevertheless, MI accompanied by an increase in ROS and leakage of cytochrome c and Ca^2+^ increases the myotoxicity of statins^324^. Hence, it is significant to explore the most appropriate dose between treatment and poisoning for the application of statins in MI.

Y-27632 is a specific inhibitor of ROCK. When used to iPSCs, it guides the differentiation of iPSCs into cardiac progenitor cells^325^, and is useful for cell therapy in cardiovascular diseases. Due to differential molecular target binding, another representative statin, atorvastatin inhibits the RhoA/ROCK pathway and its downstream molecules^326^. This may be due to RhoA non-muscle myosin II taking center stage in cell adhesion and migration^327^, which provides an important reference for future treatment of MI with drugs combined with MSCs.

## MAPK signaling pathway in MI

Mitogen-activated protein kinases (MAPKs) are a class of highly conserved serine/threonine protein kinases in cells that transmit signals through a three-level cascade. To date, four primary branches of the MAPK signaling pathway have been identified, ERK, c-JNK, p38/MAPK, and ERK5^328,329^. These kinases are sequentially activated and jointly regulate many important physiological and pathological effects, such as proliferation, growth, and differentiation of cardiac resident cells, for example, cardiomyocytes, fibroblasts, endothelial cells, and macrophages^330^. To date, many attractive inhibitors and antagonists have been developed based on the crucial role of the MAPK/ERK pathway^331,332^.

**Fig. 5:**
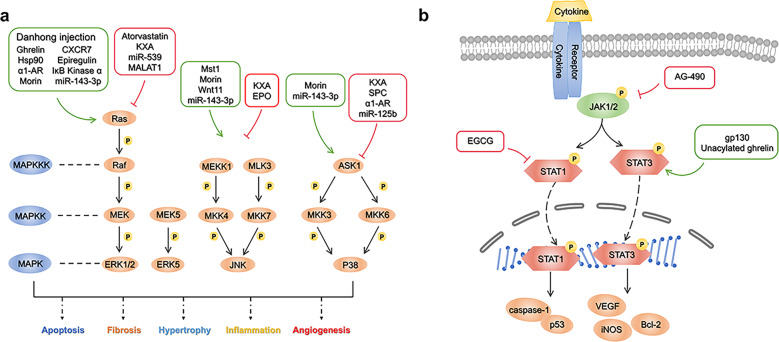
**a** MAPK signaling pathway and targeted therapy post MI following MI. MAPKs are a class of highly conserved serine/threonine protein kinases in cells that transmit signals through a three-level cascade. There are four main branches of MAPK signaling pathway, namely the ERK, the c-JNK, the p38/MAPK and the ERK5. Hsp90 Heat shock protein 90, α1-AR Alpha1 adrenergic receptor, CXCR7 CXC chemokine receptor 7, Mst1 mammalian sterile 20-like kinase 1, EPO erythropoietin. **b** JAK/STAT signaling pathway and targeted therapy following MI. JAK/STAT regulates transmembrane receptor and nuclear communication through four steps: (1) Cytokines bind to receptors, leading to dimerization of receptor molecules, and JAKs are activated and phosphorylated; (2) STAT protein is recruited to the docking site formed by these phosphorylated tyrosine sites; (3) STATs are phosphorylated and activated, which enables them to dimerize; and (4) STAT–STAT dimer translocates to the nucleus and regulates gene expression. JAK Janus kinase, STAT signal transduction and activator of transcription, EGCG epigallocatechin-3-gallate, gp130 glycoprotein 130, VEGF vascular endothelial growth factor, Bcl-2 B-cell lymphoma-2, iNOS inductible nitric oxide synthase

Although MAPK signal transduction has been well studied, the clinical efficacy of this pathway inhibitor in MI is not uniform, MAPKs’ functional mechanism and effect in MI remain to be further studied^330,333^. In this section, we mainly introduce the role of the MAPK signaling pathway in MI from the aspects of drug therapy and molecular and non-coding RNA regulation and discuss the prospects (Fig. 5a).

### Apoptosis

#### Drugs

Apoptosis is one of the most notable phenotypes mediated by the MAPK signaling pathway. Apoptosis of myocardial cells after MI leads to decreased cardiac function, while apoptosis of non-myocardial cells may aggravate the formation of cardiac scars after MI^334^. Therefore, effectively avoiding apoptosis through regulation of the MAPK signaling pathway is attractive. Some drugs target this signaling pathway. Kuanxiong aerosol inhibits myocardial injury induced by isoproterenol by inhibiting the MAPK signaling pathway^335^. The classic lipid-lowering drug atorvastatin significantly improves cardiac function and cardiomyocyte apoptosis in post-MI rats, and its mechanism is related to activation of the ERK1/2 signaling pathway^336^.

#### Molecular regulation

Some molecular effectively promote the MAPK signaling pathway and achieve regulate the phenotype of apoptosis. Wang et al. found that overexpression of Mammalian sterile 20-like kinase-1 (MST1) leads to activation of the JNK pathway, which initiates caspase-9-mediated cardiomyocyte apoptosis^337^. However, the activation of the MAPK signaling pathway is not necessarily negative, and it is widely reported that activating the ERK signaling pathway exerts a protective function in oxidative damage-induced cell death^338^. For example, ghrelin plays a cardioprotective role in mammals. It significantly reduces apoptosis after MI, and its mechanism is related to the activation of Raf-1-MEk1/2-ERK1/2 signaling pathway^339^.

Conversely, some molecules also inhibit the MAPK pathway. Erythropoietin is a glycoprotein secreted by perivascular cells in the proximal convoluted tubules of the renal cortex^340^. Studies have shown that erythropoietin reduces myocardial apoptosis after MI by inhibiting the JNK signaling pathway^341^. In addition, the regulator of G-protein signaling 5 (RGS5) is an important member of the RGS family that is closely related to cardiovascular diseases^342,343^. It was found that cardiac function in RGS5 knockout mice was significantly decreased after MI, the infarct area was significantly increased, and obvious apoptosis occurred, which may partially activate the NF-κB and MAPK signaling pathways^344^. This means that upregulation of RGS5 inhibits the MAPK signaling pathway, reducing myocardial apoptosis. Thus, based on the MAPK signaling pathway, RGS5 is a promising molecular therapeutic target.

Of note, apoptosis of non-cardiomyocytes, including myofibroblasts, after MI may aggravate myocardial remodeling and decrease cardiac function. This is because interstitial non-cardiomyocytes such as granulation tissue form scar tissue through apoptosis and death^334,345^. Therefore, blocking non-cardiomyocyte apoptosis through the MAPK signaling pathway is also a feasible method for attenuating cardiac dysfunction after MI. Sphingosylphosphorylcholine has this effect. Li et al. found that sphingosylphosphorylcholine inhibits the CaM/p38/STAT3 signaling pathway and attenuates apoptosis of cardiac myofibroblasts induced by hypoxia^334,346^.

#### Non-coding RNAs

Genomic studies based on high-throughput sequencing and microarrays also focus on the potential effects of non-coding RNAs on apoptosis after regulating the MAPK pathway^347,348^. The myocardial tissue of rats with MI infarction was injured in different degrees, and levels of miR-539 were significantly decreased. Further studies demonstrated that apoptosis and autophagy were increased after upregulation of miR-539 expression in H9C2 cells, which may be related to the targeted inhibition of MEK expression by miR-539^349^. Moreover, p38 is one of the target genes of miR-125b. It can up-regulate the expression of miR-125b, inhibits the expression of p38 and p-p38 to inhibit apoptosis^350^. In addition, lncRNA MALAT1 downregulation significantly improves myocardial function after MI in rats, which may be related to inhibition of the ERK/MAPK signaling pathway^351^.

### Fibrosis and hypertrophy

Myocardial fibrosis and hypertrophy after MI are key links of pathological ventricular remodeling that are closely related to the MAPK signaling pathway, and targeted regulation of this pathway is of great significance for improving ventricular remodeling^352,353^.

We mentioned that ANO1 attenuates post-MI myocardial fibrosis through the TGF-β/SMADs pathway^354^. However, it was been reported that ANO1 also causes fibrosis by activating the MAPK pathway^355^. We believe that the comprehensive effect of ANO1 in vivo depends on the synergistic effect of multiple pathways, which needs to be further studied. MST1 has also been reported to be associated with fibrosis and activated MST1 induces myocardial fibrosis after MI^337^. Additionally, Li et al. found that Sprouty3 was predicted to be a potential fibrosis-related target gene of miR-143-3p. MiR-143-3p promotes fibrosis through Sprouty3 degradation and downstream activation of the P38, ERK, and JNK pathways^356^.

Heat shock protein 90 is a common molecular chaperone that regulates the classic MAPK signaling pathway^357^. Tamura et al. found that heat shock protein 90 causes myocardial hypertrophy using in vitro and in vivo experiments. The mechanism may be related to increasing the stability of c-Raf in cardiomyocytes and activating the classical Raf/MEK/ERK pathway^358^. Moreover, knocking out alpha1 adrenergic receptors increased the degree of myocardial hypertrophy after MI, indicating that the deletion of Alpha1 adrenergic receptors may lead to more serious pathological myocardial remodeling in MI mouse hearts^359^.

### Inflammation

Inflammatory injury occurs in the heart after MI, and a variety of inflammatory mediators participate in the process of MI. The severity of the inflammatory reaction also determines the severity of MI, as well as the continuous pro-inflammatory response which leads to ventricular remodeling after MI^360^. The MAPK signaling pathway is correlated with the inflammatory phenotype, and targeted intervention in this pathway improves the prognosis of AMI by interfering with the occurrence and development of inflammation^361^. Duan et al. evaluated the cardioprotective effect of Osthole, an active component of Cnidium monnieri extract, in AMI. They found that Osthole improves post-MI symptoms in rats by decreasing the expression of inflammatory cytokines via activations of the MAPK pathway^362^. Morin is a bioflavonoid that resists isoproterenol-induced myocardial necrosis in rats. Results indicated that levels of proteins related to the MAPK pathway (p-JNK, P38, p-ERK1/2) and related inflammatory indices (TNF-α and IL-6) were changed, indicating that morin reduces inflammatory markers by the regulating MAPK pathway and exerts a protective effect on myocardial injury^363^. In addition, MST1 knockdown reduces inflammation and protects the heart muscle from damage after chronic infarction^337^. Erythropoietin also reduces inflammation after heart attacks^341^. Other studies regarding molecular regulation also demonstrated a correlation between the MAPK signaling pathway and inflammation; for example, low expression of RGS5 leads to activation of part of the MAPK signaling pathway and increases the occurrence of inflammation^344^. Inhibiting the expression of C-X-C chemokine receptor type 7 prevents the polarization and chemotaxis of M1 macrophages and reduces the occurrence of inflammation, which may be related to the activation of the ERK1/2 pathway^364^. In addition, miR-26b further inhibits the MAPK signaling pathway by targeting PTGS2, reducing the inflammatory response in mice after MI^365^.

### Angiogenesis

For MI, in theory, blood flow may be richer by increasing the number of blood vessels supplying ischemic tissue^366^ and targeting this pathway to promote angiogenesis could be a strategy for improving the prognosis of MI. Danhong injection is a type of traditional Chinese medicine for the treatment of cardiovascular diseases^367^. Li et al. found that after treating MI mice with Danhong injection in vivo and in vitro, the infarct area was significantly decreased, the capillary density increased, and the proliferation and migration ability of HUVECs was significantly improved. This may be related to the drug upregulating miR-126 and indirectly activating the ERK pathway^368^. Wnt is a secretory glycoprotein that plays a role in autocrine or paracrine signaling^369^. Wnt11 activates the Wnt/PKC/JNK signaling pathway, promotes angiogenesis and improves cardiac function after MI^370^. In addition, epiregulin also activates the ERK1/2 pathway and promotes angiogenesis after MI^371^. IκB Kinase α is also related to angiogenesis. Knockout of IκB Kinase α enhanced the MEK1/2/ERK1/2 pathway and reduced angiogenesis in mice after MI^372^.

### Clinical trials of the MAPK pathway in MI

In addition to the widely used statins which have a good effect on the prognosis of MI, the drugs developed in the clinic are mainly targeted at individual molecules in each branch of the MAPK pathway^373,374^. As a novel p38 MAPK inhibitor, losmapimod can effectively inhibit the expression of p38 MAPK α and β subtypes. In a phase II clinical trial, the drug effectively improved the prognosis of patients with MI and was well tolerated after oral administration^375^. But Michelle L O’Donoghue ‘s team found that although the use of losmapimod reduced the inflammatory response in patients after MI compared with placebo, it did not reduce the risk of major ischemic cardiovascular events^376,377^. Therefore, we think that the selection of Losmapimod as a therapeutic agent for patients with MI remains to be discussed, and the selection of other molecules of this pathway as therapeutic targets may be another treatment idea.

In conclusion, the MAPK signaling pathway is important due to the number of phenotypes involved. Future research should effectively promote the dominant phenotypes caused by this pathway, such as angiogenesis and inflammation reduction, and inhibit the undesirable phenotypes caused by this pathway, such as myocardial fibrosis and cardiac apoptosis. In short, fully understanding the transduction mechanism of the MAPK signaling pathway, taking this signaling pathway as the research target of MI therapy, and developing methods to improve cardiac function after MI are the keys to solving MI challenges.

## JAK/STAT signaling pathway in MI

JAK protein is a cytoplasmic tyrosine kinase associated with the intracellular domain of membrane-bound receptors^378^. Its function is to transduce signals from extracellular ligands (such as cytokines and growth factors) to the nucleus to coordinate cellular responses^378^. There are 4 members in the JAK family (JAK1, JAK2, JAK3, and TYK2) and 7 members in STAT (STAT1, STAT2, STAT3, STAT4, STAT5A, STAT5B, and STAT6)^379^. The JAK/STAT signaling pathway, also known as the IL-6 signaling pathway, is regulated by cytokines and participates in many important biological processes including cell proliferation, differentiation, apoptosis, and immune regulation^380^, which mainly regulate transmembrane receptors communicating to the nucleus^381^.

JAK/STAT regulates transmembrane receptors and nuclear communication through four steps: (1) cytokines bind to receptors, leading to dimerization of receptor molecules, and JAKs are activated and phosphorylated; (2) STAT protein is recruited to the docking site formed by these phosphorylated tyrosine sites; (3) STATs are phosphorylated and activated, which enables them to dimerize; and (4) the STAT-STAT dimer translocates to the nucleus and regulates gene expression (Fig. 5b)^382^. The JAK/STAT pathway is closely related to the occurrence and development of many diseases, such as rheumatoid arthritis^383^, Parkinson’s disease^384^, multiple sclerosis^385^, tumors, and cancer^386^. Of note, studies have shown that JAK/STAT can be used for the therapeutic intervention of cardiovascular diseases^387–391^.

### The JAK/STAT signaling regulates myocardial apoptosis

It was reported that ischemic myocardium causes cell damage to different degrees and types, and cell apoptosis is one of them^392^. Previous studies regarding the role of the JAK/STAT pathway in the cardiac tissue have primarily focused on the investigation of STAT1 and STAT3^387^. For example, the supernatant of necrotic primary cardiomyocytes (Necrotic-S) activates the JAK1-STAT1 pathway and promotes the nuclear translocation of c-Fos and NF-κB p65 after simulating the MI microenvironment, further inducing hypoxia myocardial cell apoptosis, but STAT1 silencing inhibited Necrotic-S-induced cardiomyocyte apoptosis^388^. Moreover, STAT1 reportedly also induces apoptosis in myocardial I/R by upregulating caspase-1^389^. Unlike STAT1’s pro-apoptotic effect, STAT3 exhibits an anti-apoptotic effect^390^. In the rabbit I/R model, the expression of anti-apoptotic genes BCL-2 and p-STAT3 protein significantly decreased. After injection of opioid receptors, the expression of BCL-2 and p-STAT3 increased, and the number of apoptotic cardiomyocytes decreased^391^. Furthermore, after treatment with the JAK2 inhibitor AG-490, phosphorylation of STAT3 in the myocardium of rats with MI was significantly inhibited, and the activity of caspase-3, Bax expression, and the number of apoptotic cells were significantly increased^393^. These studies indicate that the JAK/STAT pathway is closely related to the apoptotic response after MI, and STAT1 and STAT3 seem to have opposite effects.

### The JAK/STAT in angiogenesis

STAT3 plays an important role in the formation of blood vessels, and this process is essential for controlling compensatory hypertrophy and remodeling^394^. Not only that, the JAK/STAT signaling pathway also induces polarization of M2 macrophages, promoting myocardial angiogenesis and myocardial functional reconstruction^356,395^. Specific STAT3 knockout mice displayed no changes in VEGF expression, but these mice exhibited levels of VEGF inhibitors, such as thrombospondin 1 (TSP-1), and increased levels of proteins involved in the formation of interstitial matrix, such as osteopontin (OPN) and plasminogen activator inhibitor-1 (PAI-1)^394^. This leads to a pro-fibrotic and anti-angiogenic state in the heart after STAT3 is knocked out^394^. Granulocyte colony stimulating factor (G-CSF) and erythropoietin promote angiogenesis as well as improve cardiac function in MI through the JAK2/STAT pathway in a dose-dependent manner^396^. These studies demonstrated that the JAK/STAT pathway plays a crucial role in the promotion of heart remodeling via controlling angiogenesis.

### The JAK/STAT signaling as a therapeutic target for MI

Continuous activation of STAT transcription factors, especially STAT1, STAT3, and STAT5, has been described in a variety of malignant transformations^397^. Most studies indicate that STAT3 is an oncogene and that inhibiting STAT3 prevents tumor progression^398,399^, but activation of STAT3 is essential for protecting the cardiac tissue, such as by promoting angiogenesis or reducing apoptosis^390^. Therefore, in contrast to cancer treatment, the treatment of cardiovascular disease requires activation of STAT3 signaling^390^.

STAT3 is mainly activated by the interleukin (IL)-6 cytokine family, which activates glycoprotein 130 (gp130) and further causes the phosphorylation of JAK1 and STAT3^400^. Nevertheless, gp130 also activates other signal transduction cascades, including PI3k/Akt pathway^401^, as well as MAPKs (ERK1/2, JNK, p38, and ERK5)^402–404^. Of note, the differential activation of cell types and cytokines selectively activates many pathways with distinct relative activation intensities^400^. This activation can select the protective effects of these cytokines in ischemia and hypertrophic myocardium in treatment while diminishing their harmful effects^400^. In addition, many studies have shown that remote ischemic preconditioning (RIPC) reduces the area of MI and prevents I/R injury by activating JAK/STAT^405,406^. The JAK/STAT signaling may have multiple target genes, and the upregulation of the target genes may be harmful to the myocardium. For example, the STAT3 target gene iNOS induces the production of nitric oxide through IL-6 and reduces cardiac contractility^407^. Unlike STAT3 activation, STAT1 inhibitors exhibit cardioprotective effects^408^. EGCG, a STAT-1 phosphorylation and activation inhibitor, reportedly reduces infarct size and improves hemodynamic recovery and ventricular function in the I/R rat heart^408^. Hence, activation of JAK/STAT in MI should be carefully controlled, and a clear treatment strategy that balances JAK/STAT signaling should be developed to protect the heart from pathophysiological stress^390^.

Preclinical studies of the JAK/STAT signaling pathway have shown beneficial effects in preventing infarct injury after MI. Current clinical studies have also found that intracoronary perfusion mobilization of peripheral blood stem cells (PBSCs) and G-CSF in patients with myocardial infarction can improve left ventricular systolic function and remodeling, but the efficacy and safety of this study should be evaluated in a large randomized controlled trial^409^. However, we have not yet retrieved cases of the use of JAK/STAT inhibitors or agonists in human clinical trials to prevent or treat myocardial infarction. Further research is still needed to devise methods to protect the myocardium from additional damage and to aid in the management of MI.

## TGF-β/SMADs signaling pathway in MI

**Fig. 6:**
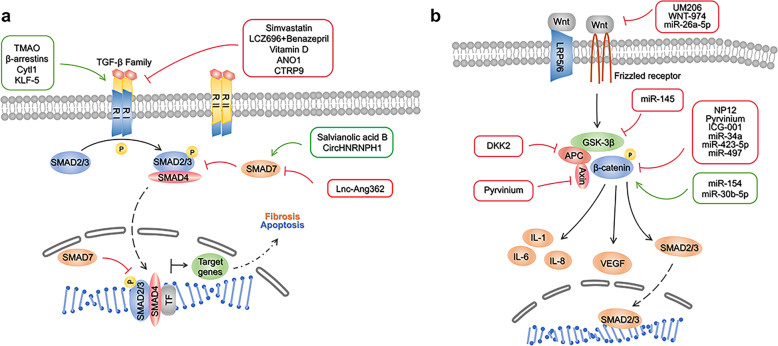
**a** TGF-β/SMADs signaling pathway and targeted therapy in MI. After TGF-β family binds to TGFβRII, TGFβRI is phosphorylated on specific serine and threonine residues, and finally forms a heterocomplex. The receptor complex reacts with the downstream effector molecule SMADs protein and eventually regulates the transcription of the target gene. RI receptors type I, RII receptors type II, TF transcriptional factor, R-Smad receptor-regulated Smad, Co-Smad common Smad, I-Smad inhibitory Smad, TMAO trimethylamine N-oxide, KLF5 Kruppel-like factor 5, Cytl1 cytokine-Like 1, ANO1 Anoctamin-1, CTRP9 C1q/tumor necrosis factor-related protein-9. **b** Wnt/β-catenin signaling pathway and targeted therapy in MI. Wnt signaling is considered as a basic growth regulation pathway. The binding of Wnt to the Frizzled receptor family and low-density lipoprotein receptor-related protein 5 (LRP5) or LRP6 co-receptors stimulates the canonical Wnt/β-catenin signaling pathway, thereby regulating the stability of β-catenin and context-related transcription. Wnt wingless, LRP LDL receptor-related protein, APC adenomatous polyposis coli, GSK-3β glycogen synthase kinase 3β, VEGF vascular endothelial growth factor, SMAD small mother against decapentaplegic, IL interleukin, DKK2 Dickkopf-related protein 2

At present, the human TGF-β family includes several members, such as TGF-β, bone morphogenetic protein (BMP), growth and differentiation factor (GDF), activin, inhibin, and so on^410^. After the TGF-β family binds to TGFβRII, TGFβRI is phosphorylated on specific serine and threonine residues and finally forms a heterocomplex^411,412^. The receptor complex reacts with the downstream effector SMADs protein and eventually regulates the transcription of the target gene^413^. At present, there are 8 kinds of SMADs, which are divided into 3 classes according to their functions, namely, receptor-regulated SMAD (R-SMAD), common SMAD (Co-SMAD), and inhibitory SMAD (I-SMAD)^414^. The TGF-β complex binds to R-SMADs and Co-SMADs to form heteromers, translocates into the nucleus in an R-SMAD-Co-SMAD complex, and transduces specific signals to regulate the transcription of target genes to exert its biological effects^415^ (Fig. 6a).

This signaling pathway is related to the occurrence and development of different diseases, including tumors, tissue fibrosis, cardiovascular diseases as well as rheumatic immune diseases^416–418^. Researchers have found that this pathway also plays a crucial role in myocardial fibrosis, apoptosis, and other pathological processes after MI^419^. Therefore, there are an increasing number of studies targeting this pathway for the treatment of MI^22,420^.

### TGF-β/SMADs and myocardial fibrosis after MI

Previous studies have shown that the TGF-β/SMADs signaling pathway plays a critical role in tissue repair^421^. TGF-β1 is closely related to post-MI and ventricular remodeling and is one of the most important factors promoting myocardial fibrosis^422^. To date, recombinant TGF-β1 protein is widely used to establish the fibrosis model in vitro^423–425^.

Cardiac fibroblasts secrete the pro-fibrosis cytokine TGF-β1 and activate the TGF-β1/SMADs signaling pathway after MI^426^. Moreover, TGF-β1 and downstream SMAD2/3/4 expression are increased to varying degrees in the infarct area and the infarct boundary area^427^. In addition, TGF-β1 promotes the transformation of cardiac fibroblasts into myofibroblasts. Expression of α-SMA, a hallmark of mature myofibroblasts, was significantly increased in the infarct boundary area^428,429^. Myofibroblasts further release related inflammatory factors, angiotensin-II, and other cytokines that promote fibrosis, further aggravating cardiac fibrosis^429,430^. It is worth noting that in the early stage of MI, increased expression of TGF-β1 promotes the recruitment of fibroblasts to the infarction site and secretion of collagen and other substances to promote the recovery of myocardial injury^431^; however, continued fibrotic responses cause cardiac remodeling and reduced heart function, which eventually lead to heart failure^426^. Therefore, it is of great significance to explore the positive and negative effects of TGF-β and effectively regulate the expression of key molecules in this pathway for the development of new therapeutic strategies for myocardial fibrosis^432,433^.

#### Drugs

Due to the critical role of the TGF-β/SMADs signaling in MI, many inhibitors and antagonists have been developed. Recent studies have found that simvastatin can downregulate TGF-activated kinase 1, reducing TGF-β expression, and improving ventricular remodeling^434^. Notably, the antihypertensive drug valsartan significantly decreased the expression of TGF-β/SMADs, HIF-1α, and fibrosis-related proteins in rats after MI and significantly improved the cardiac function, infarct size, wall thickness, and myocardial vascularization in ischemic hearts^304^. Liu’s group showed that the combination of LCZ696 (an angiotensin receptor-neprilysin inhibitor) and benazepril (an angiotensin-converting enzyme inhibitor) exerts a good positive regulatory effect on myocardial fibrosis after MI in mice, and its mechanism was also closely related to the decrease in TGF-β1^435^. Besides conventional inhibitors and antagonists, additional vitamin D supplementation and aerobic resistance training also regulate the expression of collagen type I and III by downregulating the TGF-β1/SMAD2/3 signaling pathway, further improving cardiac function and alleviating cardiac fibrosis^436^.

Additionally, several active components of traditional Chinese herbs may also have anti-fibrosis effects through this pathway. Salvianolic acid B, an effective component of Salvia miltiorrhiza, reduces myocardial collagen fibers, decreases the expression of TGF-β1 and SMAD2/3, and increases expression of SMAD7 in vivo and in vitro, which ultimately improves fibrosis^437^. In addition, it was found that tanshinone IIA reduces the expression levels of collagen type I and III, TGF-β, α-SMA, MP2, and MMP9 in myocardial infarcted rats and angiotensin-induced cardiac fibroblasts^438^. Yu et al. also found that Ginsenoside Re may improve cardiac dysfunction induced by MI and reduce ventricular remodeling by regulating the AMPK/TGF-β1/SMAD2/3 signaling pathway^439^.

#### Molecular regulation

Through the application of gene therapy (gene silencing, gene knockout, gene overexpression, etc.), chemical reagents, and recombinant proteins, key molecules in this pathway can be regulated, thus affecting the occurrence and development of MI^440,441^. Trimethylamine N-oxide is an intestinal microbial metabolite that is reported to be relevant to the poor prognosis of ischemic heart disease. It activates the TGF-βRI/SMAD2 pathway and aggravates excess cardiac fibrosis and dysfunction after MI^442^. β-Arrestins are the signaling molecules involved in the desensitization of β-adrenergic receptors. Upregulation of β-Arrestins in cardiac fibroblasts after MI promotes the transformation of fibroblasts to myofibroblasts and collagen synthesis stimulated by TGF-β^443^. In addition, cytokine-like 1 may aggravate myocardial fibrosis after MI by activating the TGF-β/SMADs signaling pathway^444^.

Notably, some molecules can also reduce fibrosis by negatively regulating this pathway. For example, ANO1 is a calcium-activated chloride channel protein in human cardiac fibroblasts. In in vivo and in vitro experiments, the degree of cardiac fibrosis was decreased after overexpression of ANO1^354^. C1q/tumor necrosis factor-related protein-9 has been found to reverse ventricular remodeling and effectively reduce visceral fibrosis via the SMAD2/3 signaling pathway^445^. Moreover, Nogo-C protein and exogenous BMP-7, which can inhibit this pathway, have also been reported to reduce fibrosis and improve ventricular remodeling^446,447^. Overexpression of the Notch1 intracellular domain antagonizes TGF-β 1-induced SMAD3 phosphorylation and alleviates the occurrence of fibrosis^107^. Besides, Notch3 has been found to have similar effects^448^. Therefore, it may be a promising method for Notch signal activators and TGF-β/SMADs signaling inhibitors to be used for the treatment of fibrosis after MI.

#### Non-coding RNAs

In recent years, studies regarding non-coding RNAs have emerged and a growing number of findings have demonstrated that the non-coding RNAs play very important roles in regulating the TGF-β/SMADs signaling pathway^449–451^. Accordingly, it was found that miR-195 promotes fibrosis in MI rats upregulating TGF-β1/SMADs pathway^452^. Downregulating the expression of miR-130 upregulates the expression of peroxisome proliferator-activated receptor γ and indirectly inhibits TGF-β1, suppressing cardiac fibrosis^453^. In addition, including but not limited to MALAT1, CircRNA 010567, miR-133a and miR-224 have also been found to affect cardiac remodeling after MI by regulating this pathway^454–457^.

Of note, some non-coding RNAs directly target key molecules of this pathway to play a regulatory role in MI. For example, SMAD7 is not only the I-SMAD of the TGF-β/SMADs signaling pathway but is also the direct target of Lnc-Ang362. Upregulation of Lnc-Ang362 directly suppressed the expression of SMAD7, promoted the expression of this pathway, and aggravated fibrosis after MI^458^. In addition, SMAD7 is the direct target of miR-216-5p, and overexpression of miR-216-5p aggravates the occurrence of fibrosis. CircHNRNPH1, a sponge of miR-216-5p, downregulates the expression of miR-216-5p and indirectly upregulates the expression of SMAD7, attenuating reactive fibrosis^459^.

#### Cell therapy

Cell therapy is seen as a promising clinical approach, and the application of BMMSCs is a kind of cell therapy that improves cardiac function after MI^460^. Wei et al. found that ultrasound targeted microbubble destruction-mediated galactose lectin-7-small interfering RNA therapy enhanced the homing ability of BMMSCs, inhibited TGF-β1/SMADs signaling pathway activation and reduced fibrosis after MI^461^. Hypoxic preconditioned MSCs reduce the activation of fibroblasts by secreting leptin, which may involve inhibition of the TGF-β/SMAD2 signaling pathway^462^. In addition, MSC transplantation combined with pioglitazone improves myocardial remodeling through the TGF-β1/SMADs signaling pathway^463^.

### The role of the TGF-β/SMADs signaling pathway in apoptosis after MI

The TGF-β/SMADs signaling pathway mediates multiple phenotypes, which not only plays a role in tissue repair but also apoptosis^464^. After MI, continuous ischemia and hypoxia will lead to activation of TGF-β, which leads to high expression of SMAD2/3, resulting in apoptosis of cardiomyocyte, and further aggravating myocardial injury^392,465^. The reduction in cardiomyocyte apoptosis during MI is beneficial for the improvement of cardiac function; therefore, targeting this pathway and regulating pericardial apoptosis are particularly important.

There are few studies on the treatment of apoptosis based on the TGF-β/SMADs pathway in the field of MI, and in recent years, it has mainly focused on the regulation of this pathway by ncRNAs. Kruppel-like factor 5 (KLF5) promotes apoptosis in cardiomyocytes and it has been found that KLF5 may activate the TGF-β/SMAD2/3 signaling pathway by downregulating miR-27a, resulting in cardiomyocyte injury after MI^466^. MiR-808 downregulates the expression of TGF-β1, inhibits the expression of caspase-3 and caspase-9, and inhibits cardiomyocyte apoptosis^465^. Exocrine bodies derived from ADSCs contain miR-671, which reduces cardiomyocyte apoptosis by inactivating the TGFβRII/SMAD2 axis^467^. Moreover, LncRNA SOX2-OT aggravates hypoxia-induced cardiomyocyte injury by regulating the miR-27a-3p/TGFβRI axis^468^. In addition, downregulation of circRNA 010567 expression improves cardiac function and inhibits myocardial apoptosis. The mechanism may be related to inhibition of the TGF-β signaling pathway^454^.

In most cases, apoptosis is not beneficial in the heart after MI, regardless of whether it occurs in cardiomyocytes or non-cardiomyocytes^334^. However, TGF-β/SMADs are a double-edged sword, and prematurely targeting inhibition of this pathway to inhibit apoptosis inevitably affects tissue repair in the early stage of MI. Briefly, much more work should be done on the development of new therapeutics targeting the TGF-β/SMADs signaling pathway.

### Clinical trials of the TGF-β/SMADs pathway in MI

The statin, angiotensin converting enzyme inhibitor (ACEI) and angiotensin receptor antagonist (ARB) mentioned above that may inhibit this pathway have been widely used in clinical practice and achieved good results in patients with MI^469,470^. Besides, N-acetylcysteine (NAC) can reduce serum TGF-β levels in Patients with ST-segment elevation MI^471^. In addition, short-term use of Sodium Tanshinone IIA can also effectively reduce left ventricular remodeling in MI patients^472^. However, there is still a lack of inhibitors or agonists of the TGF-β/SMADs signaling pathway in large-scale clinical trials, coupled with the two-sidedness of this pathway in the process of cardiac obstruction, further research is needed on the effects of targeted drugs and timing of drug use on patients with MI.

## Wnt/β-catenin signaling pathway in MI

The Wnt signaling pathway is related to the development process and affects the cell cycle at various time points^473^. Simply put, Wnt is a growth stimulating factor that causes cell proliferation^474^. At the same time, it acts as a directional growth factor in the process of tissue growth^475–477^. In the field of developmental evolution and cancer therapy, Wnt signaling has been considered as a basic growth regulation pathway^473^. It is divided into two categories: β-catenin-dependent signaling (canonical pathway) and β-catenin-independent signaling (non-canonical pathway)^478^. Binding of Wnt to the Frizzled receptor family and low-density lipoprotein receptor-related protein 5 (LRP5) or LRP6 co-receptors stimulates the canonical Wnt/β-catenin signaling pathway, thereby regulating the stability of β-catenin and context-related transcription^479^. On the other hand, the transmembrane receptor Tyr kinases Ror2 and Ryk and Frizzled receptors that act independently of LRP5 or LRP6, activate the non-canonical Wnt pathway^479^. This pathway drives cell movement^480^ and changes in polarity^481^.

Increasing evidence has shown that Wnt signaling is triggered during the pathological process of MI injury (Fig. 6b). Studies have demonstrated that Wnt activation is related to pathological stages after MI, including inflammation, angiogenesis, and fibrosis^482^. Analysis of the expression of Wnt proteins indicated that Wnt-2, Wnt-4, Wnt-10b, and Wnt-11 were significantly upregulated 5 days after MI^483^. The researchers used Axin2-LacZ to express LacZ in cells with active typical Wnt signaling, demonstrating that Wnt signaling is activated in cardiomyocytes located in the border zone of the infarct^484^. In the TopGAL mouse model expressing the marker β-gal under the control of TCF/LEF1, an increase in Wnt signaling activity was detected 4 days after MI^483^.

### The Wnt/β-catenin signaling pathway and inflammation in MI

The repair of infarct myocardium includes three stages: inflammation, proliferation, and maturity. Inflammation is first activated in MI^172^. Wnt-5a promotes the release of IL-1, IL-6, and IL-8 from monocytes, indicating that it has a pro-inflammatory effect^485^. β-catenin-mediated signals are activated in pro-inflammatory macrophages after MI, which is manifested by increased lymphocyte infiltration levels and increased expression of pro-inflammatory cytokines^486^. In addition, another study reported that the absence of Wnt inhibitory factor 1 (WIF1) causes increased inflammatory monocytes and severe adverse remodeling, while overexpression of WIF1 weakens the monocyte response and improves cardiac function^487^.

### The Wnt/β-catenin signaling pathway and angiogenesis in MI

Angiogenesis manifests as newly formed blood vessels by endothelial cells, which is conducive to heart repair and functional recovery after MI^482^. A previous study showed that the Wnt signaling pathway is located in the cytoplasm of the vascular endothelium during the neovascularization process after MI, which is reflected by the accumulation of β-catenin^488^. In fact, many negative Wnt modulators have been shown to promote angiogenesis in the heart after MI^482^. Overexpression of the FrzA/sFRP-1 gene increases capillary density in MI scars through the inhibition of Wnt signaling^489^. Likewise, Dickkopf2 (DKK2), another Wnt inhibitor, stimulates endothelial cell angiogenesis after MI via LRP6/APC activation^490^. Nevertheless, one study also found that the allosteric inhibitor NP12 stabilizes β-catenin and activates the Wnt signaling pathway, which in turn promotes angiogenesis and improves ventricular function after MI^491^.

### The Wnt/β-catenin signaling pathway and cardiac fibrosis in MI

Cardiac remodeling is regarded as a key determinant of the clinical outcome in heart disease and cardiac fibrosis is a major aspect of the remodeling process^492^. Myocardial fibrosis is an important pathophysiological process observed after MI^493^. Studies have shown that the Wnt/β-catenin signaling pathway plays a major role in the regulation of cardiac fibrosis^494^. Interestingly, TGF-β signaling also interacts with the Wnt signaling pathway and plays a key role in the differentiation of myofibroblasts^492^. Regarding the interaction between the Wnt and TGF-β signaling, studies have demonstrated that Wnt3a can up-regulate TGF-β signaling through the canonical β-catenin-dependent Wnt signaling of SMAD2, inducing myofibroblast differentiation^495^. In acute ischemic heart injury, the upregulates Wnt1 is initially expressed in the epicardium and then expressed by cardiac fibroblasts in the injured area^496^. Wnt1 induces cardiac fibroblasts to proliferate and express pro-fibrosis genes^496^. Except for the role of Wnt, the absence of β-catenin in cardiac fibroblasts alleviates pressure-overload-induced fibrosis in mice, preserves cardiac function, and reduces interstitial fibrosis^497^. In addition to research on signaling molecules, based on the results of Cui et al.^498^, miR-145 also reduces heart fibers by directly targeting SOX9 in fibroblasts and regulating the Akt/GSK-3β/β-catenin signaling pathway change. This shows that miRNAs can also inhibit cardiac fibrosis after MI.

### The Wnt/β-catenin pathway as a therapeutic target for MI

Since Wnt/β-catenin plays a critical role in MI, the development/use of Wnt/β-catenin inhibitors has been attractive for MI therapy. Pyrvinium, a Wnt inhibitor, was successfully used to stabilize β-catenin and inhibit Axin degradation^499^. An increase in Ki-67^+^ cells was observed in the peri-infarct and distal myocardium of animals treated with pyrvinium, which reduced adverse cardiac remodeling^499^. ICG-001, a β-catenin inhibitor, inhibits the β-catenin signaling pathway and reduces the expression of S100A4, alleviating cardiac fibrosis in mice, indicating that S100A4 may be a therapeutic target for cardiac fibrosis^500^. Due to differences in target binding, UM206 is a selective frizzled protein antagonist, which inhibits Wnt/Frizzled signaling and was used to reduce the expansion of infarct size and prevent the development of heart failure^501^. In addition, WNT-974 improves the recovery of heart function after ligation of the left anterior descending coronary artery by reducing the undesirable remodeling of the infarct tissue^482^. Its mechanism involves preventing the production of collagen in cardiomyocytes by blocking the secretion of Wnt3 (a pro-fibrotic agonist) from cardiac fibroblasts and its signal transmission to cardiomyocytes^502^. These studies indicate that Wnt pathway inhibitors are a class of potential drugs that treat MI through many mechanisms, including increasing angiogenesis, inhibiting fibrosis, and stimulating heart regeneration.

In recent years, the role of non-coding RNA in MI has emerged. One study found that miR-26a-5p targets WNT5A to inhibit the activity of the Wnt/β-catenin signaling pathway, inhibit H/R-induced cardiomyocyte damage and apoptosis, and restore cell viability^467^. However, additional studies have observed that miRNAs activate the Wnt pathway to promote the development of MI, and their inhibitors may be more therapeutic. For example, miR-30b-5p promotes myocardial cell apoptosis in rats with MI by activating the Wnt/β-catenin signaling pathway^503^. MiR-154 has the same effect as miR-30b-5p^504^. MiR-34a inhibitors^505^ and miR-423-5p inhibitors^506^ reduce apoptosis and cardiomyocyte damage after MI in rats by activating the Wnt/β-catenin signaling pathway to improve cardiac function. From the above studies, the roles of non-coding RNA in the Wnt pathway are not entirely the same, and understanding these differences may require further research.

Cell therapy has been extensively tested to restore heart function after MI^88,89^. Cardiac progenitor cells induced by human induced pluripotent stem cells using cardiogenic small molecules effectively regenerate the infarcted heart and reduce fibrosis, and can target a variety of genes related to cardiac differentiation signaling pathways, including Wnt, cytoskeleton remodeling, and TGF-β induced epithelial mesenchymal transition (EMT) signal, VEGF^507^. Another study found that the coordinated angiogenesis of cardiac MSCs and direct induction of TGF-β/Wnt signals in MSCs in the myocardium initiate an accelerated healing process and promote heart recovery^508^. In addition, activation of the Akt/GSK3β/β-catenin signaling axis helps cortical bone-derived stem cells (CBSCs) to play an important protective role in the myocardium by reducing the area of MI, improving cardiac function, and increasing capillary density^509^. Interestingly, a study found that miR-497 inhibitors activate the Wnt/β-catenin pathway to promote the effects of BMMSCs transplantation in the treatment of MI^510^.

At present, treatment of MI based on the Wnt/β-catenin signaling pathway has been verified in many animal experiments, but animal models cannot fully replicate all the processes that occur after human MI, so the results of preclinical studies should be carefully explained^511^. Many current clinical studies have found that Wnt/β-catenin targeted drug therapy or stem cell therapy are more widely used in various cancer patients. Two clinical phase I studies have shown that the Wnt/β-catenin pathway is related to the inhibitors CWP232291^512^ and OMP-18R5^513^ can improve the occurrence of adverse events. However, in the research of myocardial infarction, drug therapy and stem cell therapy have been fully explored in preclinical research, and there is still a lack of clinical research to further transform and verify the important role of the Wnt/β-catenin signaling pathway in the prevention and treatment of myocardial infarction. Future research may begin with drugs that have been shown to target Wnt signaling in diseases such as cancer to further test the benefits of intervening in Wnt signaling in cardiovascular disease. These experiments are likely to shed more light on the feasibility and benefits of targeting Wnt signaling in cardiovascular disease.

## Hippo signaling pathway in MI

**Fig. 7:**
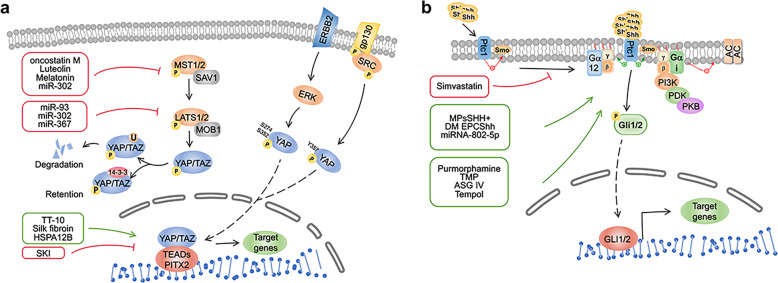
**a** Hippo/YAP signaling pathway and targeted therapy after MI. Canonical and noncanonical Hippo pathways are vital mechanisms of homeostasis, repair, and regeneration in the heart. In canonical Hippo/YAP, when the pathway turns “on”, the activated MST, SAV, LATS, and MOB leads phosphorylated YAP/TAZ detained in the cytoplasm or degraded, progressively. In contrast, the pathway turns “off” up-regulating the coaction of YAP/TAZ and other transcription factors. MST1/2 mammalian sterile 20-like kinases 1/2, SAV1 salvador family protein 1, LATS1/2 large tumor suppressors 1/2, MOB1A/1B Mps one binder kinase activator-like 1A/1B, YAP Yes-associated protein, TAZ PDZ-binding motif. **b** Sonic Hedgehog signaling pathway and its correlated intervention after MI. Shh Sonic Hedgehog, Ptc Patched, Smo smoothened, Gli glioma-associated oncogene homolog, TMP tetramethylpyrazine, AGS astragaloside

Because the Hippo pathway has been implicated in regulating organ size and tissue homeostasis^514,515^, there is infinite interest in uncovering the regulatory mechanism of the Hippo pathway in MI^29^. As an evolutionarily conserved signaling pathway, the key components in mammals include MST1/2, Salvador family protein 1 (SAV1), large tumor suppressors (LATS1/2), Mps one binder kinase activator-like 1A/1B (MOB1A/1B), Yes-associated protein (YAP), and PDZ-binding motif (TAZ), which maintain high consistency with *Drosophila*^516,517^. In response to microenvironmental cues, Hippo kinase MST1/2 heterodimerizes with SAV1, and consequently phosphorylates LATS1/2 and the coactivator MOB1, in turn activating the coordinated ubiquitination and 14-3-3 binding of phosphorylated YAP and TAZ, finally suppressing their nuclear localization and degradation^29,518^ (Fig. 7a).

### The Hippo pathway in cardiomyocyte regeneration after MI

Evidence from the latest study showed that MI induces regional patterns of cycling cardiomyocytes^519^. Since studies have found that the Hippo pathway plays an important role in homeostasis of the cardiovascular system, by controlling cardiomyocyte proliferation and survival^29^, it has been suggested that there may be tremendous potential for targeting the Hippo pathway for therapeutic intervention in MI^520^. Moderate loss of function of the Hippo component is a desirable strategy for alleviating cardiac injury in MI^521^. Among these molecules, MST1 works as a forward modulating regulator in cardiac dysfunction induced by ischemia^522^. Suppressing the activation of MST1 mainly mitigates adverse cardiac remolding and relieves heart dysfunction^523,524^. Additionally, *Sav* was found to be inversely associated with cardiac function and angiogenesis, and positively related to cardiac fibrosis^525,526^; promotion of LATS2 is deemed to be a negative mediator in cardiomyocyte proliferation^527^. In the nucleus, activation of transcriptional effector YAP/TAZ, either by inactivation of Hippo kinase cascade components, or by forced activation of YAP/TAZ in a Hippo-independent manner, is desirable for cardiomyocyte renewal therapy. When non-phosphorylated YAP and TAZ enter the nucleus, they bind to transcription cofactors, such as TEA domain transcription factor (TEAD)^528^ and paired-like homeodomain 2 (PITX2)^529^, to activate target cardiomyocyte protection genes^514^. Based on experimental discoveries, YAP activation induces cardiomyocytes to re-enter the myocyte cycle and proliferate in both fetal and adult mouse hearts^519^, and likewise, overexpression of YAP1 mediated by adeno-associated virus (AAV9) alleviates injury and improves the heart function^530^.

Besides the canonical Hippo pathway, numerous studies have focused on the molecular mechanisms of Hippo components in cardiac regulation post injury. Epigenetics, Han et al. pointed out the involvement of α-ketoglutarate-dependent dioxygenase alkB homolog 5 (ALKBH5)-mediated m^6^A demethylation promotes the translation of YAP, consequently, leading to the promotion of cardiomyocyte proliferation, reduction of infarct size, and marked the restoration of cardiac function^531^. The beneficial effect of gene therapy with constitutive AAV-gp130 has been demonstrated to promote the proliferation of cardiomyocytes by activating macrophage recruitment via the Hippo-independent Src-Yap pathway^532^. Heart regeneration following MI is regulated by an intricate network of signaling cascades, and signaling between and within cells is highly complex^533^. Since research has gradually focused on exploring the mechanism, by means of high-throughput sequencing, the synergistic effect on cardiac recovery has been reflected^534,535^. Multiple signaling pathways including PI3K/Akt, BMP-SMAD1/5, Hippo/YAP, and MAPK/ERK, are all controlled via lysophosphatidic acid_3_ (LPA_3_) mediation to enhance cardiac function and heart regeneration^534^, and the EMT-like regenerative response is regulated by ERBB2-mediated YAP^535^. These results revealed that the independent Hippo pathway regulates transcriptomics and proteomics in cardiomyocyte regeneration during injury.

### The Hippo pathway mediates inflammation, fibrosis, and angiogenesis following cardiac injury

In addition to the myocardium responding to the occurrence of infarction through the Hippo pathway, the pericardium, inflammatory cells, cardiac fibroblasts, and vascular endothelial cells play an essential role in regulating cardiac function through this pathway during the recovery phase^29,536,537^. Deleted *Yap* and *Taz* in the adult murine epicardium resulted in defective regulatory T cell infiltration following tamoxifen-induced injury, leading to cardiac fibrosis, cardiomyopathy, and a high rate of mortality, along with pro-fibrotic F4/80^+^ macrophages recruitment^538^. Moreover, during the progression of MI, the exaggerated fibrotic response, in general, leads to progressive heart failure. Recent studies have investigated whether the Hippo pathway plays a unique role in regulating fibroblast state transitions. LATS1/2 and YAP are required for maintaining cardiac fibroblasts in a resting state and myofibroblast differentiation; hence, deletion of *Lats1/2* or inhibition of YAP limits the YAP-dependent inflammation and fibrogenesis response to injury^539,540^. Admittedly, prolonged fibrogenesis contributes to scar expansion and heart failure^9^, and effective interventions to prevent or reverse cardiac fibrosis are urgently needed. Although SMAD/TGF-β signaling is commonly regarded as the core regulator in cardiac fibrosis, it has been shown that SKI also triggers the Hippo pathway and deactivates TAZ to inhibit myofibroblast activation^541^. In contrast, angiogenesis is generally encouraged to prevent heart failure after MI. The heat shock protein (HSPA12B) in endothelial cells cooperates with YAP to regulate the process of vascular remodeling^542^.

### Therapeutic strategies for MI based on the Hippo pathway

As a potential contributor to the regulation of cardiac regeneration, inflammatory, fibrotic, and angiogenic phenotypes, the Hippo signaling pathway is considered a desirable target for treatment. Except for the studies focusing on gene therapy mentioned in the previous section^525,530,532,534^, administering a high dose of AAV9-*Sav*-short hairpin RNA (AAV9-*Sav*-shRNA) directly into border zone cardiomyocytes revealed a mild improvement in the ejection fraction of pig heart^526^, similar to findings in mouse model from Leach’s group^525^.

Apart from studies that have demonstrated the feasibility of gene therapy, the newest studies have focused on other interventions, for instance, drug therapy, cell therapy, and therapies based on biomaterials, exosomes, and non-coding RNAs. Drug intervention for suppressing activation of MST1 might represent a promising strategy for cardiac protection^522^. The cardioprotective effects of oncostatin M^523^, luteolin^543^, and melatonin^544^ have been verified by observing enhanced cardiomyocyte autophagy and mitochondrial biogenesis in MI by targeting MST1. Moreover, to extend the duration of pharmaceutical drug delivery, Chen et al. encapsulated, the fluorine substituent of TAZ-12, TT-10, into polylactic-co-glycolic acid nanoparticles, which effectively activated the cell cycle of hiPSC-CMs and inhibit apoptosis by upregulating YAP^545^. Feng et al. implanted reduced graphene oxide (rGO)/silk fibroin-modified nanofibrous biomaterials into the heart, showing a direct effect on preventing rat ventricular remodeling via YAP/TAZ^546^. Due to the regenerative properties of stem cells, stem cell therapy has been engaged to repair injured heart tissues^547^. Intriguingly, with the aid of exosome biocompatibility, human cardiac explant-derived progenitor cells (CPCs)-derived exosomes carried the extracellular matrix protein periostin to regulate the cardiomyocyte proliferation^548^. Remarkably, non-coding RNAs including miRNAs have been demonstrated to sufficiently induce cardiomyocyte proliferation and regeneration^549,550^. In a recent study, high-content miRNA screening of hiPSC-CMs confirmed the core node of the Hippo pathway in controlling cardiomyocyte proliferation as a potential miRNA target^551^. In particular, miR-93, miR-302, and miR-367 attenuate cardiac remodeling by targeting *LATS2*^527,552,553^, *Mst1*, and *Mob1b*^553^ and promote angiogenesis^552^ after MI. Of interest, although transduction of non-coding RNAs can be achieved by gene therapy using AAVs or small nucleic acids, and delivery of biomaterial nanoparticles or engineering exosomes, enveloping non-coding RNAs could facilitate their delivery to the damaged myocardium with high efficiency and safety^554^.

Although current clinical trials of cardiac regenerative therapies have encountered obstacles, revealing limitations and difficulties in translating preclinical experiments into the clinic, there are still several studies aiming to overcome this bottleneck^555,556^. Pioneer showed the cardioprotective effects of melatonin, acting as a suppressor of MST1^544^, administered in patients with ST-segment elevation MI (STEMI) after primary percutaneous coronary intervention^162^.

Undoubtedly, the molecules of Hippo signaling components are potential target spots for cardiac disease treatment. However, extensive experiments focused on these therapeutic strategies converge on the Hippo pathway in large mammal preclinical models and high-quality clinical trials are still required to advance toward clinical application.

## Sonic hedgehog signaling pathway in MI

Hedgehog was discovered in 1980 by Nusslein-Volhard and Wieschaus to regulate the polarity of Drosophila segments^557^. There is only one Hh gene in fruit flies, while mammals have three: Sonic Hedgehog (Shh), Indian Hedgehog (Ihh), and Desert Hedgehog (Dhh)^558^. All three Hh gene-secreted proteins exhibit catalytic capacity. Shh is the most widely distributed in human tissues and cells, participating in gene transcription, regulating the expression of cytokines and functional proteins^558^, and playing an extremely important role in regulating embryonic growth and development, angiogenesis, and tumor cell proliferation^559^.

The Sonic hedgehog signaling pathway is composed of the signaling molecule hedgehog, patch receptor Ptc (Patched), Smoothened (Smo), and Glioma-associated oncogene homolog (Gli)^560,561^. Unlike other growth and development signaling pathways, the Sonic hedgehog signaling pathway is highly dependent on a single organelle, the primary cilia^560,561^. The cilia are packed with proteins required for Sonic hedgehog signal transduction, the important signal components are concentrated in a small area on the tip of the cilia to achieve an effective response, and their distribution on the cilia changes according to the presence or absence of Sonic hedgehog signals^562^. The Sonic hedgehog pathway has a pleiotropic effect in alleviating cardiac ischemic injury by improving angiogenesis and recruiting EPCs^560,561^, protecting myocardial cells by decreasing apoptosis and oxidative stress^19^, and reducing the occurrence of reperfusion arrhythmia^563^ (Fig. 7b). Previous studies used a Sonic hedgehog agonist to activate the Sonic hedgehog pathway^564^, and they found that coronary artery density was increased and coronary artery function was also improved after MI^564^. Accordingly, blocking the Sonic hedgehog signal of cardiomyocytes reduced the expression of coronary angiogenic genes and the number of vessels^564^.

### The Sonic hedgehog pathway in promoting angiogenesis after MI

During myocardial ischemia, expression levels of Sonic hedgehog and Ptc increased, which promotes bone marrow-derived EPCs to induce angiogenesis and promotes the expression of angiogenic factors^565^. Studies have indicated that Sonic hedgehog gene therapy regulates angiogenesis by activating the Sonic hedgehog pathway^565^, improves the speed and quantity of coronary angiogenesis^566,567^, and then increases coronary blood supply which further improves cardiac function^566^. Previous studies also used a Sonic hedgehog agonist to activate the Sonic hedgehog pathway^564^, and they found that coronary artery density was increased and coronary artery function was also improved after MI^564^. Accordingly, blocking the Sonic hedgehog signal of cardiomyocytes reduced the expression of coronary angiogenic genes and the number of vessels^564^.

Many studies have demonstrated the mechanism of Sonic hedgehog signaling in promoting angiogenesis^560,561,565,567,568^. First, the Sonic hedgehog pathway increases the number of EPCs and promotes their function. The Sonic hedgehog pathway recruits EPCs to the site of myocardial ischemia, promotes neovascularization, inhibits myocardial fibrosis, and prevents myocardial apoptosis in a chronic myocardial ischemia model^565^. Another study used microparticles to carry Sonic hedgehog morphogen (MPShh+) and EPCs to improve vascular regeneration and found that MPShh+ increased the angiogenesis of EPCs and the production of NO^567^. Second, overexpression of Sonic hedgehog in endothelial cells increased VEGF to regulate angiogenesis^560,561^. During ischemia and hypoxia, increased expression of Sonic hedgehog and Ptc not only promotes the induction of angiogenesis by bone marrow-derived EPCs but also promotes expression of angiogenic factors, including angiopoietin and VEGF, in cardiac microvascular endothelial cells^560,561^. Third, the function of endothelial cells could be promoted by the Sonic hedgehog pathway^568^. It has also been suggested that the hedgehog signaling pathway, as the target gene of platelet-derived growth factor-BB (PDGF-BB), upregulates ERK1/2 and phosphorylates Akt, playing a role in the migration and recruitment of vascular endothelial cells^568^.

Considering the differential mechanisms of Sonic hedgehog pathways on angiogenesis, researchers have used multiple methods to improve angiogenesis after MI, such as stem cell therapy and pharmacological compounds^569–571^. Some studies injected Sonic hedgehog modified-CD34+ cells into the edge of acute MI in mice and found that the infarct size was significantly reduced^569,570^. Additionally, the activation of BMMSCs in Sonic hedgehog pathways induced angiogenesis and endogenous cardiac regeneration through paracrine effects^571,572^. Another study used erythropoietin to induce Sonic hedgehog signaling to repair the heart after MI^573^. Consistent with this, the activation of hedgehog signaling in the adult heart leads to an increase in coronary vessel density^574^. These studies implicate Sonic hedgehog signaling as an essential regulator of coronary vascular development and as a potential therapeutic target for coronary heart diseases. Further studies should explore whether the Sonic hedgehog signaling-induced angiogenesis has therapeutic value in MI.

### Activation of the Sonic hedgehog pathway decreases cardiomyocyte apoptosis

It was reported that increased survival and decreased apoptosis of cardiomyocytes enhance the repair of myocardial function^564^. Activation of the Sonic hedgehog pathway increases the survival rate of cardiomyocytes and reduces apoptosis caused by myocardial ischemia^575^. Sonic hedgehog also promotes the recovery of left ventricular function by decreasing programmed myocardial cell death^564^. This study also demonstrated that downregulating the Sonic hedgehog signaling of cardiomyocytes leads to apoptosis and dysfunction of cardiomyocytes^564^. Based on the role of the Sonic hedgehog pathway in myocardial apoptosis, previous studies used different compounds including miRNAs^576^, the agonists and antagonists of Sonic hedgehog^2,577^, and cell therapy, to explore the mechanism of Sonic hedgehog in apoptosis^2^. One study suggested that silencing miR-802-5p targets PTCH1 and activates the Sonic hedgehog signaling pathway to inhibit apoptosis and reduce myocardial injury after MI^576^. Adding the Sonic hedgehog signaling pathway receptor agonist to oxygen glucose deprivation (OGD)-induced myocardial cells downregulated the expression of Bcl-2 and Bax, and decreased the number of apoptotic cells. Nevertheless, the administration of the antagonist SANT-1 had the opposite effect^2^. In addition, in a diabetic myocardial ischemia model, autologous cell therapy using diabetic EPCs suppressed myocardial apoptosis and improved angiogenesis, thus reducing cardiac fibrosis and finally restoring myocardial function through the Shh/Bmi1/p53 axis^578^.

### The Sonic hedgehog pathway in decreasing oxidative stress

Besides the role of the Sonic hedgehog on cardiomyocytes and apoptosis, activation of the Sonic hedgehog pathway also reduces oxidative stress after MI. Many agonists and antagonists of the Sonic hedgehog have been developed to explore their role in oxidative stress. One study reported that purmorphamine, a Sonic hedgehog agonist, prevents the ovariectomized heart from myocardial injury by attenuating the expression of TNF-α and MPO levels and the release of LDH and CK-MB^577^. However, silencing the effects of Shh using cyclopamine, a specific inhibitor of Shh, or siRNA, an inhibitor of the Shh receptor Patched, strongly reduced the production of NO^579^. These studies suggest the potential role of Sonic hedgehog in the decrease in oxidative stress. Furthermore, another study used antioxidative strategies and found that it could reactivate the endogenous Sonic hedgehog pathway and contribute to myocardial healing as well as the improvement of diabetic cardiac function^580^. Based on its antioxidative role, researchers have applied new methods to decrease injury of oxidase stress. Microparticles (MPs) reportedly carry molecules in the Sonic hedgehog pathway to induce expression of NO and decreases the production of reactive oxygen species^579^. Injection of MPs also improved endothelial function by decreasing oxidase stress injury^579^.

### The Sonic hedgehog pathway increases autophagy after MI

Studies have shown that autophagy plays an important role after MI and that activating autophagy could represent a new therapeutic method for cardiac protection^581^. Up to now, few studies have reported the role of the Sonic hedgehog pathway in activating autophagy. The main mechanism was correlated with the AMPK pathway^19,579^. The Sonic hedgehog pathway promotes the phosphorylation of the AMPK pathway and combines with it to induce autophagy^579^. One study added SAG to H9C2 cardiomyocytes with OGD and found that SAG stimulated autophagy and promoted H9C2 cardiomyocyte survival, and they suggested that the Sonic hedgehog pathway protected cardiomyocytes through an AMPK-dependent autophagy^19^. In addition, inhibition of autophagy using AMPK inhibitor also weakened the protective effect of Sonic hedgehog on myocardial cell autophagy after infarction^19^.

### The controversial role of the Sonic hedgehog pathway in myocardial I/R injury

Although most studies have reported the protective role of Sonic hedgehog pathways in MI^582^, there are only a few studies have reported the opposite results in the model of myocardial I/R injury^583,584^. One study proposed that Sonic hedgehog had no cardio-protective effect on cardiomyocytes after myocardial I/R injury^583^. They found that the overexpression of Sonic hedgehog in human stem cell-derived cardiomyocytes did not increase vascularization of the infarct scar^583^. Another study even suggested that the Sonic hedgehog pathway plays a detrimental role in myocardial repair. They found that simvastatin decreased myocardial I/R injury by inhibiting the Sonic hedgehog pathway^584^. The opposite results of the Sonic hedgehog pathway may be explained by the different models (MI and I/R models) used in previous studies. The permeant MI model and myocardial I/R injury model may induce slightly different scars and lead to slightly different repair mechanisms which may change how the tissue responds to Sonic hedgehog signaling.

### The Sonic hedgehog pathway in clinical applications

Since Sonic the hedgehog pathway has critical roles in promoting myocardial repair, it may serve as a potential cardiac therapeutic target^565,585^. Sonic hedgehog gene therapy may have considerable therapeutic potential in individuals with acute and chronic myocardial ischemia by triggering the expression of multiple trophic factors and engendering tissue repair in the adult heart^565^.

The first application is microparticles (MPs), which are small fragments generated from the plasma membrane after cell stimulation. A previous study activated the Sonic hedgehog pathway by N-Shh or shed membrane microparticles harboring Sonic hedgehog ligand (MPs (Shh+)) to protect the heart from I/R injury by preventing the occurrence of arrhythmias^563^. Secondly, it can be applied in gene targeted therapy^578,582,586^. There are many methods to activate the expression of Sonic hedgehog in cardiomyocytes, including recombination of Sonic hedgehog proteins, using microparticles loaded with Sonic hedgehog, knocking out Patched genes, injection of Sonic hedgehog mRNA, as well as the Sonic hedgehog receptor agonists^585^. These methods could improve the motility of smooth muscle cells, induce the migration of smooth muscle cells, recruit parietal cells into neovascularization, upregulate VEGF and angiopoietin, increase the number of capillaries and promote cardiac vascular maturation after MI^578^, reduce myocardial cell apoptosis^578^, inhibit left ventricular remodeling^578^, increase the number of myocardial cells^582^, and improve cardiac function after MI^585^. Thirdly, the application in comprehensive therapy. The Sonic hedgehog signaling pathway promotes cardiac function by upregulating angiogenic genes and enhancing the mobilization of bone marrow-derived progenitor cells. Combination therapy using PHSHH and AMD3100 effectively stimulates progenitor cell mobilization, improves capillary density, and reduces myocardial fibrosis to enhance cardiac function recovery^575^. Lastly, some drugs, including tetramethylpyrazine and astragaloside IV were reported to preserve cardiac function after MI by upregulating Sonic hedgehog, Smo, and Gli-2^587^. Tempol reduced oxidative stress to restore the endogenous Shh pathway and improve diabetic cardiac function^580^.

Some clinical trials have explored the potential therapeutic effect of CD34+ cells^588^, BMMSCs^589^, and erythropoietin^590–592^ in coronary heart disease. Although some of these clinical trials show application prospects, they are not widely used in clinical practice. Further studies should explore whether these cell therapy and drugs of activating Sonic hedgehog signaling-induced angiogenesis has therapeutic value and could be safely and effectively applied in patients with MI.

## Conclusion and perspectives

Ischemic heart disease has become a serious threat to human life and health, therefore novel therapeutic strategies for the treatment of MI are in urgent need. Over the past decades, the developed therapeutic strategies have taken into consideration the impact of the cellular and molecular levels in MI pathological processes as well as the treatment procedures. Herein, most of the current strategies in MI therapy show promising clinical application prospects in the recovery of MI such as pharmacotherapy, gene therapy, protein therapy, cell therapy, as well as exosome therapy. It is evident that the preclinical experiments and clinical experiments targeting molecular signaling following myocardial ischemia have achieved promising effects. In this review, we comprehensively highlighted and summarized the most relevant signaling pathways involved in MI treatment (Table 1).

**Table 1:** Intervention of signaling pathways in MI

Therapeutic strategy	Diseases (Model)	Target pathway	Intervention	Author/Year
Drug	MI	PI3K/Akt/mTOR	Everolimus (mTOR inhibitor)	Buss et al. 2009^65^
	TAC	eNOS	L-NAME (eNOS inhibitor)	Kazakov et al. 2013^39^
	I/R	PI3K/Akt/GSK-3β	Kaempferide	Wang et al. 2017^41^
	Langendorff system	PTEN/PI3K/Akt	VO-OHpic	Li et al. 2018^63^
	MI	PI3K/Akt/mTOR	TNP (IP7 inhibitor)	Deng et al. 2019^46^
	MI/H_2_O_2_	mTOR	Tanshinone IIA	Zhang et al. 2019^70^
	MI	PTEN/PI3K/Akt	HOpic (PTEN Inhibitor)	Feng et al. 2020^61^
	MI	mTOR	Rapamycin	Chen et al. 2021^47^
	MI	PI3K/Akt/mTOR	Ivabradine	Dai et al. 2021^67^
	MI/SGD	mTOR	Sphingosine-1-phosphate	Yang et al. 2021^69^
	MI	PI3K/Akt/VEGF	Gastrin	Fu et al. 2021^605^
	MI	Notch	TNF-α inhibitor	Pei et al. 2015^125^
	MI	Notch	Melatonin	Pei et al. 2016^145^
	MI	Notch	Yiqihuoxue prescription	Wu et al. 2017^143^
	MI	Notch	Astragaloside	Yu et al. 2017^144^
	MI	Notch	Ligusticum Chuanxiong Radix Paeonia	Shi et al. 2019^140^
	MI	Notch	Pigment epithelium-derived factor	Liu et al. 2019^159^
	MI	Notch	Oestrogen Receptor β	Du et al. 2020^158^
	I/R	Notch	ALDOA	Luo et al. 2020^126^
	MI	TLR4	Metformin	Soraya et al. 2014^241^
	MI	NF-κB	Methotrexate	Maranhão et al. 2017^242^
	I/R	NF-κB, AP-1	Hemin	Yeh et al. 2009^266^
	MI	HO-1, connexin-43	Cobalt protoporphyrin	Kusmic et al. 2014^267^
	MI	EET/HO-1	Agonists of EETs	Cao et al. 2015^268^
	MI	NRF2/HO-1	Wogonin loaded NPs	Bei et al. 2020^263^
	MI	KEAP1/NRF2/HO-1	Hirudin	Zhang et al. 2020^264^
	MI	NRF2/HO-1 TLR4/TNF-α	Dapsone	Abdelzaher et al. 2021^226^
	MI	PI3K/Akt/Nrf2/HO-1	Rosuvastatin combined with low-dose carvedilol	Baraka et al. 2021^265^
	MI	RhoA	Rosuvastatin	Bulhak et al. 2007^322^
	MI	RhoA/ROCK	Coptisine	Gong et al. 2012^319^
	MI	RhoA/ROCK	Estradiol	Lee et al. 2014^306^
	MI	RhoA/ROCK	Ibuprofen	Patel et al. 2016^320^
	MI	RhoA/ROCK	Nicorandil	Lee et al. 2018^307^
	MI	RhoA/ROCK	Fasudil	Zhou et al. 2020^321^
	MI	RhoA/ROCK	Fluvastatin	Yi et al. 2020^323^
	MI	RhoA/ROCK	Dexmedetomidine	Sun et al. 2021^318^
	MI	MAPK	Osthole	Yeung et al. 2018^361^
	MI	MAPK	Atorvastatin	Zeng et al. 2019^336^
	MI	MAPK	Danhong injection	Li et al. 2019^368^
	MI	MAPK	KXA	Lu et a. 2021^335^
	MI	Wnt/β-catenin	Pyrvinium	Saraswati et al. 2010^499^
	MI	Wnt/β-catenin	Wnt antagonist Dickkopf2	Min et al. 2011^490^
	MI	Wnt/β-catenin	Aldehyde dehydrogenase-2	Zhao et al. 2015^494^
	MI	Wnt/Frizzled	UM206	Uitterdijk et al. 2016^501^
	MI	Wnt/β-catenin	NP12	Baruah et al. 2017^491^
	MI	Wnt/β-catenin	ICG-001	Qian et al. 2018^500^
	MI	TGF-β2, TGF-β3	Fasudil	Hattori et al. 2004^312^
	MI	TGFβ1/TAK1	Fasudil	Li et al. 2012^311^
	MI	TGF-β/SMADs	Valsartan	Sui et al. 2015^304^
	MI	TGF-β/SMADs	Simvastatin	Xiao et al. 2016^434^
	MI	TGF-β/SMADs	Salvianolic acid B	Gao et al. 2019^437^
	MI	TGF-β/SMADs	Ginsenoside Re	Yu et al. 2020^439^
	MI	TGF-β/SMADs	LCZ696 + benazepril	Liu et al. 2021^435^
	MI	TGF-β/SMADs	Vitamin D + ART	Mehdipoor et al. 2021^436^
	MI	TGF-β/SMADs	Tanshinone IIA	Chen et al. 2021^438^
	MI	JAK/STAT	IL-33	Li et al. 2019^395^
	MI	JAK/STAT	Hyaluronic acid Oligosaccharides	Lee et al.2019^606^
	MI	Hippo/YAP	Luteolin	Hu et al. 2016^543^
	MI	Hippo/YAP	Melatonin	Hu et al. 2017^544^
	STEMI	Hippo/YAP	Melatonin Adjunct	Dominguez-Rodriguez et al. 2017^162^
	MI	Hippo/YAP	oncostatin M	Yang et al. 2018^522^
	MI	YAP	TT-10-delivered NPs	Chen et al. 2021^545^
	MI	Sonic Hedgehog	Erythropoietin	Ueda et al. 2010^573^
	MI	Sonic Hedgehog	Tempol	Xiao et al. 2015^580^
	MI	Sonic Hedgehog	Tetramethylpyrazine Astragaloside IV	Wang et al. 2017^587^
	MI	Sonic Hedgehog	Purmorphamine	Sharma et al. 2018^577^
	MI	Sonic Hedgehog	Simvastatin	Feng et al. 2020^584^
Gene therapy	MI	PI3K/Akt/FOXO3a	Ab-NGF Ad-human NGF	Meloni et al. 2010^58^
	MI	PTEN/PI3K/Akt	Ad-PTEN	Parajuli et al. 2012^64^
	MI	mTOR	lenti-miR-99a	Li et al. 2014^81^
	MI	PI3K/Akt/FOXO	Period 2 KO EPC	Sun et al. 2014^91^
	MI	PTEN/PI3K/Akt	miR-130a	Lu et al. 2015^77^
	MI	Akt	Ad-GHSR-1a GHSR-1a siRNA	Yuan et al. 2016^32^
	MI	PTEN/PI3K/Akt	miR-21 miR-146a	Huang et al. 2016^75^
	MI	PTEN/PI3K/Akt	EnMSCs-Exo-miR-21	Wang et al. 2017^76^
	MI	Akt/PDGF‐D	Akt‐hucMSCs-Exo PDGF‐D siRNA	Ma et al. 2017^90^
	MI	PI3K/p-Akt	AAV9-activated PDGFR-β	Yue et al. 2019^31^
	MI	PTEN/PI3K/Akt	sh-GAS5 miR-142-5p inhibitor	Du et al. 2019^82^
	MI	PTEN/PI3K/Akt	sh-AZIN2-sv	Li et al. 2019^85^
	OGD	PI3K/Akt/mTOR	pEX-UCA1	Zhang et al. 2019^86^
	Chronic MI	Akt/FoxO3a	AAV9-SERCA2a	Kumarswamy et al. 2012^74^
	MI	PTEN/PI3K/Akt	PTEN cKO mice	Liang et al. 2020^62^
	MI	PTEN/PI3K/Akt	Senescent MSCs-Exo miR-221-3p	Sun et al. 2020^78^
	MI	PTEN/PI3K/Akt	miR-301a	Zhen et al. 2020^79^
	MI	PTEN/PI3K/Akt	sh-GAS5 OE-GAS5 miR-21 mimics	Zhou et al. 2020^83^
	Hypoxia	PI3K/Akt/mTOR	pcDNA3.1-DANCR	Qiu et al. 2020^87^
	MI	PTEN/PI3K/Akt	lncRNA Snhg1 Snhg1 cKO mice	Li et al. 2021^80^
	MI	PI3K/Akt	sh-CircHIPK3 miR-93-5p agomiR	Wu et al. 2021^84^
	MI	Notch	miR-199b	Chen et al.^148^
	MI	Notch	miR-429	Xu et al. 2016^154^
	MI	Notch	miR-363	Meng et al. 2017^135^
	MI	Notch	miR-208a	Zhang et al. 2018^123^
	MI	Notch	miR-1	Chen et al. 2018^153^
	MI	Notch	CYP2J2	Zhao et al. 2018^161^
	MI	Notch	miR-29b	Liu et al. 2019^120^
	MI	Notch	miR-374	Zhao et al. 2019^152^
	MI	Notch	KCNQ1OT1	Wang et al. 2019^155^
	MI	Notch	lncRNA XIST	Zhang et al. 2019^156^
	MI	Notch	KRT1	Fang et al. 2019^160^
	MI	Notch	miR-384-5p	Fan et al. 2020^112^
	MI	Notch	CircRNA Hipk3	Si et al. 2020^147^
	MI	Notch	miR-29b-3p	Yang et al. 2020^149^
	MI	Notch	miR-124a	Xu et al. 2021^146^
	MI	Notch	miR-133	Zhang et al. 2021^150^
	MI	Notch	miR-106a-363	Jung et al. 2021^151^
	MI	P2X7/NLRP3	Brilliant blue G	Vessey et al. 2010^202^
	MI	NLRP3	16673-34-0	Marchetti et al. 2015^193^
	MI	NLRP3	MCC950	van Hout et al. 2017^194^
	MI	NLRP3	JC124	Fulp et al. 2018^197^
	MI	NLRP3	OLT1177	Toldo et al. 2019^198^
	MI	NLRP3	Oridonin	Gao et al. 2021^196^
	MI	TLR4	TAK-242	Fujiwara et al. 2019^240^
	MI	TLR4	ApTOLL	Ramirez-Carracedo et al. 2020^238^
	I/R	HO-1	AAV-HO-1	Melo et al. 2002^260^
	I/R	HO-1	AAV2-HO-1	Liu et al. 2007^259^
	MI	TLR4	Radioprotective 105 (RP105)	Louwe et al. 2014^239^
	MI	TLR4	lenti-shRNA	Liu et al. 2015^243^
	MI	β-MHC, ANF, BNP	NRF2 KO	Strom et al. 2017^17^
	MI	RhoA/ROCK/HIF-1α	Notch3 siRNA	Shi et al. 2020^121^
	MI	MAPK	α1-AR KO	Yeh et al. 2017^359^
	MI	MAPK	miR-539	Hui et al. 2017^349^
	MI	MAPK	Mst1KO	Wang et al. 2018^337^
	MI	MAPK	RGS5 KO	Ding et al. 2018^344^
	MI	MAPK	ghrelin	Eid et al. 2019^339^
	MI	MAPK	EPO	Li et al. 2019^341^
	MI	MAPK	17-AAG (Hsp90 inhibitor)	Tamura et al. 2019^358^
	MI	MAPK	Morin	Verma et al. 2019^363^
	MI	MAPK	Epiregulin siRNA	Cai et al. 2019^371^
	MI	MAPK	IκB Kinase α KO	Cao et al. 2019^372^
	MI	MAPK	miR-26b	Ge et al. 2019^365^
	MI	MAPK	miR-143-3p	Li et al. 2019^356^
	MI	MAPK	MALAT1	Fan et al. 2019^351^
	MI	MAPK	SPC	Li et al. 2019^346^
	MI	MAPK	lenti-ANO1-RNAi	Tian et al. 2020^355^
	MI	MAPK	CXCR7 shRNA	Zhang et al. 2020^364^
	MI	MAPK	pMSCV-EGFP-Wnt11	Wang et al. 2020^370^
	MI	MAPK	miR-125b	Qiao et al. 2020^350^
	MI	Wnt/β-catenin	Transgenic Mice OE FrzA	Barandon et al. 2003^489^
	MI	Wnt/β-catenin	miR-34a antagomir	Li et al. 2019^505^
	I/R	Wnt/β-catenin	miR-423-5p inhibitor	Zhu et al. 2019^506^
	MI	Wnt/β-catenin	miR-30b-5p inhibitor	Chi et al. 2020^503^
	MI	Akt/GSK‐3β/β‐catenin	Ad‐miR‐145	Cui et al. 2021^498^
	I/R	Wnt/β-catenin	miR-26a-5p	Yan et al. 2021^467^
	MI	TGF-β/SMADs	Cytl1 KO	Kim et al. 2016^444^
	MI	TGF-β/SMADs	Notch3 siRNA Notch3 cDNA	Zhang et al. 2016^448^
	MI	TGF-β/SMADs	Ad-ANO1-GFP	Gao et al. 2017^354^
	MI	TGF-β/SMADs	Exogenous BMP-7	Jin et al. 2018^447^
	MI	TGF-β/SMADs	Nogo-C KO Nogo-C-shRNA	Weng et al. 2018^446^
	MI	TGF-β/SMADs	miR-130	Chu et al. 2018^453^
	MI	TGF-β/SMADs	TMAO	Yang et al. 2019^442^
	MI	TGF-β/SMADs	β-arrestins siRNA	Philip et al. 2019^443^
	MI	TGF-β/SMADs	Ad‐CTRP9 shCTRP9	Liu et al. 2019^445^
	MI	TGF-β/SMADs	Ad‐N1ICD/Smad3 Ad‐shN1ICD/Smad3	Zhou et al. 2019^107^
	MI	TGF-β/SMADs	miR-133a	Yu et al. 2019^456^
	MI	TGF-β/SMADs	miR-224	Xu et al. 2019^457^
	MI	TGF-β/SMADs	MALAT1	Huang et al. 2019^455^
	MI	TGF-β/SMADs	miR-195	Wang et al. 2020^452^
	MI	TGF-β/SMADs	miR-808	Zhang et al. 2020^465^
	MI	TGF-β/SMADs	Lnc-Ang362	Chen et al. 2020^458^
	MI	TGF-β/SMADs	LncRNA SOX2-OT	Yang et al. 2020^468^
	MI	TGF-β/SMADs	CircRNA 010567	Bai et al. 2020^454^
	MI	TGF-β/SMADs	KLF5-specific inhibitor ML264	Tian et al. 2021^466^
	MI	TGF-β/SMADs	miR-671	Wang et al. 2021^467^
	MI	TGF-β/SMADs	CircHNRNPH1	Li et al. 2021^459^
	I/R	JAK/STAT	EGR-1	Mudaliar et al. 2017^406^
	MI	JAK/STAT/c-Fos	miR-181 miR-150	Zhu et al. 2017^388^
	I/R	JAK/STAT	Unacylated ghrelin (UAG)	Sawashita et al. 2020^405^
	MI	Hippo/YAP	Tg-DN-Mst1	Odashima et al. 2007^524^
	MI	Hippo/YAP	lenti-miR302-367 OE pLKO.1-Yap shRNA	Tian et al. 2015^553^
	MI	Hippo/YAP	AAV9-SAV-shRNA SAV cKO mice	Leach et al. 2017^525^
	hiPSCs	Hippo/YAP	miR-302d OE	Xu et al. 2019^607^
	MI	Hippo/YAP	LPA_3_-KO mice AAV9-LPA_3_-OE	Wang et al. 2020^534^
	MI	Hippo/YAP	lenti-miR-93 OE	Ma et al. 2020^552^
	MI	Hippo/YAP	AAV9-SAV-shRNA	Liu et al. 2021^526^
	MI	Hippo/TAZ	Ad-SKI	Landry et al. 2021^541^
	MI	YAP/TAZ	YAP cKO mice	Ramjee et al. 2017^538^
	MI	YAP	AAV9-human YAP	Lin et al. 2014^530^
	MI	YAP	AAV-gp130	Li et al. 2020^532^
	MI (HF)	YAP	caERBB2-OE mice pAAV-CMV-YAP	Aharonov et al. 2020^535^
	MI	YAP	YAP cKO mice	Francisco et al. 2020^540^
	MI	YAP	HSPA12B cKO mice YAP cKO mice	Fan et al. 2020^542^
	MI	YAP	AAV9-ALKBH5-KO	Han et al. 2021^531^
	MI	YAP	hCPC-Exo carried periostin	Balbi et al. 2021^548^
	MI	Sonic Hedgehog	Shh-AMD3100	Roncalli et al. 2011^575^
	MI	Sonic Hedgehog	MSCs	Tang et al. 2013^571^
	MI	Sonic Hedgehog	PEG hydrogel	Johnson et al. 2015^586^
	MI	Sonic Hedgehog	MPsSHH+	Paulis et al. 2015^582^
	MI	Sonic Hedgehog	DM EPCShh	Xiao et al. 2019^578^
	MI	Sonic Hedgehog	MPsSHH+	Bueno-Betí et al. 2019^567^
	MI	Sonic Hedgehog	MPsSHH+	Ghaleh et al. 2020^563^
	MI	Sonic Hedgehog	miR-802-5p	Li et al. 2021^576^
Cell therapy	Hypoxia	Akt	Akt-MSCs	Gnecchi et al. 2005^92^
	MI	Akt	Akt-MSCs	Gnecchi et al. 2006^95^
	MI	Notch	Embryonic stem cell	Tsang et al. 2017^134^
	MI	Notch	MSCs CSCs	Shevchenko et al. 2019^141^
	MI	Wnt/β-catenin	hiPSCs CPCs ISX-9	Xuan et al. 2018^507^
	I/R	Akt/GSK3β/β-Catenin	Cortical bone-derived stem Cell Trop2	Li et al. 2018^509^
	MI	TGF-β/SMADs	MSCs+pioglitazone	Chen et al. 2014^462^
	MI	TGF-β/SMADs	Hypoxic preconditioned MSCs	Hou et al. 2015^463^
	MI	TGF-β/SMADs	BMMSCs	Wei et al. 2021^461^
	MI	JAK/STAT	G-CSF- and erythropoietin-based cell therapy	Kang et al. 2008^396^
	MI	Sonic Hedgehog	MSCs (Shh)	Ahmed et al. 2010^572^
	MI	Sonic Hedgehog	CD34 (Shh)	Mackie et al. 2012^569^
	MI	Sonic Hedgehog	hiPSCs	Munarin et al. 2020^608^
Exosome therapy	MI	PTEN/PI3K/Akt	Explant-derived cardiac stromal cells-Exo	Qiao et al. 2019^102^
	MI	Sonic Hedgehog	PAMsHGF	Riaud et al. 2021^609^
Protein therapy	MI	PI3K/Akt	Recombinant FLT3 Ligation	Pfister et al. 2014^73^
	MI	YAP/TAZ	rGO/Silk Fibroin-Modified Nanofibrous Patches	Feng et al. 2021^546^
*Combination therapy*				
Drug Cell therapy	H/SD	PI3K/Akt	EGb761 BMMSCs	Li et al. 2011^97^
Drug Gene therapy	HFD MI	mTORC1	Rapamycin Ad-Rheb	Sciarretta et al. 2012^51^
Drug Gene therapy	MI	PI3K/Akt/mTOR	PI3Kγ KO mice AAV9-PRAS40 Insulin	Völkers et al. 2013^45^
Drug Gene therapy	MI	eNOS	L-NAME (eNOS inhibitor) human HSPA12B OE mice	Li et al. 2013^43^
Drug Cell therapy	MI	PI3K/Akt/ FOXO3a	Rosuvastatin; AD-MSCs	Zhang et al. 2013^100^
Drug Cell therapy	MI	Akt	TNP (IP6Ks inhibitor) MSCs	Zhang et al. 2014^99^
Drug Cell therapy	MI	Akt	Edaravone BMMSCs CSCs	Zhang et al. 2016^96^
Cell therapy Gene therapy Exosome therapy	MI	PI3K/Akt/mTOR	MSCs SDF1 OE Exo-SDF1	Gong et al. 2019^103^
Protein therapy Cell therapy	MI	Akt	Thymosin β4 hiPSC-CMs	Tan et al. 2021^93^
Protein therapy Cell therapy	MI	PI3K/Akt	NGF hucMSCs	Luo et al. 2021^94^
Gene therapy Cell therapy	MI	Akt	lenti-TMSB4 BMMSCs	Tang et al. 2021^98^
Gene therapy Cell therapy	MI	HO-1	MSCs OE HO-1	Zeng et al. 2008^272^
Gene therapy Cell therapy	MI	HO-1	MSCs OE HO-1	Zeng et al. 2010^270^
Gene therapy Cell therapy	MI	HO-1	MSCs OE HO-1	Jiang et al. 2011^271^
Drug Cell therapy	MI	RhoA/ROCK/ERK	Atorvastatin	Zhang et al. 2014^326^
Drug Cell therapy	MI	TGF-β/Wnt	Epicardial erythropoietin Stem cells	Klopsch et al. 2018^508^
Gene Cell therapy	MI	Wnt/β-catenin	miR-497 antagomir BMMSCs	Tang et al. 2019^510^

*AAV* adeno-associated virus, *Ab-NGF* nerve growth factor-neutralizing antibody, *Ad* adenoviral virus, *AD-MSCs* adipose-derived mesenchymal stem cells, *AZIN2-sv* lncRNA-AZIN2 splice variant, *CD34(Shh)* Sonic hedgehog-modified human CD34+ cells, *cKO* conditional knockout, *CSCs* cardiac stem cells, *DM* EPCShh adenovirus Shh-modified diabetic EPCs, *EETs* epoxyeicosatrienoic acids, *EnMSCs* human endometrium-derived mesenchymal stem cells, *EPC* endothelial progenitor cell, Exo exosome, *FLT3* FMS-like tyrosine kinase 3, *G-CSF* granulocyte colony stimulating factor, *GAS5* growth arrest-specific transcript 5, *GHSR-1a* growth hormone secretagogue receptor1a, *gp130* glycoprotein 130, *H/SD* hypoxia/serum deprivation, *H*_*2*_*O*_*2*_ hydrogen peroxide, *hCPCC* human cardiac explant-derived progenitor cells, *HF* heart failure, *HFD* high fat diet-induced obesity and metabolic syndrome, *hiPSCs* human-induced pluripotent stem cells, *hiPSC-CMs* human-induced pluripotent stem cell-derived cardiomyocytes, *HSPA12B* heat shock protein A12B, *hucMSCs* human umbilical cord mesenchymal stem cells, *I/R* ischemia and reperfusion, *IP6Ks* inositol hexakisphosphate kinases, *IP7* inositol pyrophosphates, *lenti* lentivirus, *lncRNA* long non-coding RNA, *L-NAME* L-N(G)-nitroarginine methyl ester, *MI* myocardial infarction, *miRNA* microRNA, *MPsSHH+* shed membrane microparticles harboring SHH ligand, *MSCs* mesenchymal stem cells, *NPs* nanoparticles, *OE* overexpression, *OGD* oxygen-glucose deprivation, *PAMsHGF* pharmacology active microcarriers encapsulated hepatocyte growth factor, *PDGF-D* platelet‐derived growth factor D, *PEG* biodegradable polyethylene glycol, *Rheb* Ras homology enriched in brain, *SDF1* stromal-derived factor 1, SGD serum-and glucose-deficient, *shRNA* short hairpin RNA, *Shh* Sonic hedgehog, *siRNA* small interfering RNA, *Snhg1* small nucleolar RNA host gene 1, *STEM* ST-segment elevation myocardial infarction, *TAC* transverse aortic constriction, *Tg-DN-Mst1* overexpression of dominant negative Mst1, *TMSB4* thymosin β4 gene, *TNP* N6-(*p*-nitrobenzyl) purine, *UCA1* urothelial carcinoma associated 1

**Fig. 8:**
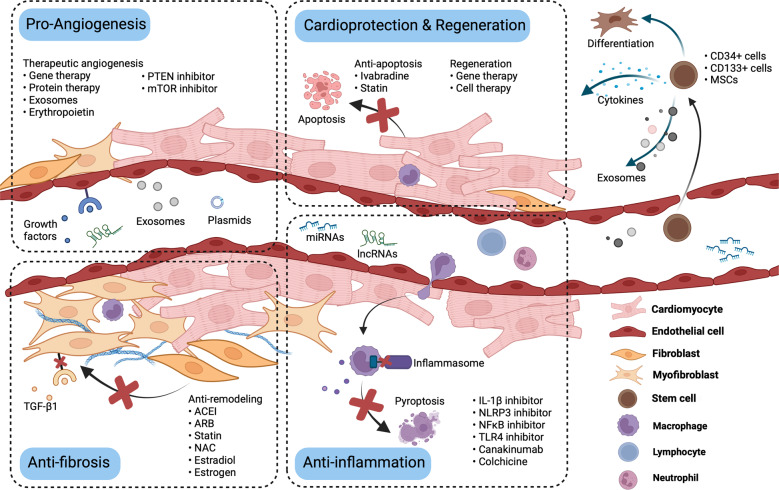
Novel targeted therapeutic strategies and mechanisms in MI treatment (Created with BioRender.com). In terms of the signaling pathways, potential therapeutic strategies for MI that have been proposed to include drug, gene therapy, protein therapy, cell therapy, and exosome therapy. According to the pathological process of MI, the specific targeted mechanism of these therapies could be classified into four categories: (1) anti-inflammation, (2) anti-fibrosis, (3) cardioprotection and cardiac regeneration, and (4) pro-angiogenesis. Dot-labeled subtitles refer to the representative targeting drugs or bioactive molecules. ACEI angiotensin converting enzyme inhibitor, ARB angiotensin receptor antagonist, EPCs endothelial progenitor cells, lncRNA long non-coding RNA, miRNA microRNA, mTOR mammalian target of rapamycin, NAC N-acetylcysteine, NLRP3 nucleotide-binding domain, leucine-rich-repeat family, pyrin-domain-containing 3, PTEN phosphatase and tensin homolog, TGF-β1 transforming growth factor-β1, IL-1β interleukin-1β, TLR4 Toll-like receptor 4, NF-κB nuclear factor-κB, MSCs mesenchymal stem cells

**Fig. 9:**
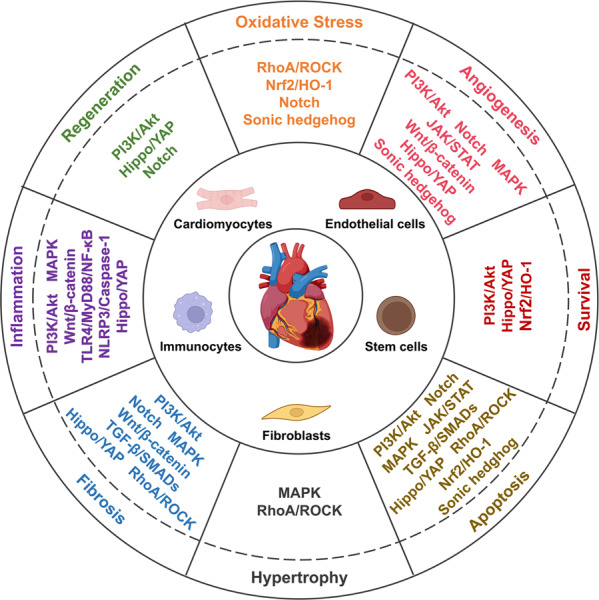
Cell signaling pathways participant in regulating pathological processes and phenotypes after MI (Created with BioRender.com). PI3K/Akt phosphoinositide-3 kinase/protein kinase B, TGF-β/SMADs transforming growth factor-β/SMADs, Wnt/β-catenin wingless/β-catenin, NLRP3/caspase-1 nucleotide-binding domain, leucine-rich-repeat family, pyrin-domain-containing 3/caspase-1, TLR4/MyD88/NF-κB toll-like receptor 4/MyD88/Nuclear factor-κB, Nrf2/HO-1 nuclear factor erythroid derived 2-related factor 2/heme oxygenase-1, MAPK mitogen-activated protein kinase, JAK/STAT Janus kinase/signal transducer and activator of transcription, Hippo/YAP Hippo/Yes-associated protein

It is well-established that the damage of cardiac tissue caused by ischemia-hypoxia is a composite result of the cellular change in response to stimuli, in addition, these cells also participate in cardiac repair and regeneration following MI^10,360,392^. It follows from the above that cardiac protection and functional restoration can be achieved through a multi-targeted approach, which modulates the flow of cellular signals in different indigenous or migrated cells. Herein, in order to describe the pivotal role of signaling pathways in the biological process of MI vividly, we diagramed the fundamental signaling pathways in cardiomyocytes, endothelial cells, fibroblasts, monocytes, as well as (myeloid or transplanted) stem cells, in the pathological changes and the treatment of MI (Figs. 8, 9). Principal signaling pathways mentioned here include the PI3K/Akt, Notch, TGF-β/SMADs, Wnt/β-catenin, NLRP3/caspase-1, TLR4/MyD88/NF-κB, Nrf2/HO-1, RhoA/ROCK, MAPK, JAK/STAT, Hippo/YAP, and Sonic hedgehog pathways, which mainly centered on various pathological states such as inflammation, oxidative stress, fibrosis, hypertrophy, apoptosis, survival, angiogenesis, and regeneration post MI (Fig. 9). Remarkably, these pathways form a complex and homeostatic regulating network, rather than act in isolation. In this context, it should be emphasized that the novel therapies which mediate crosstalk pathways may exert more beneficial effects in cardiac repair and secondary prevention of MI.

In the preclinical studies for MI treatment, the potential effect of drug, gene therapy, and cell therapy on MI point out the promising direction of clinical research. The drugs, such as Ivabradine, colchicinef, canakinumab, rapamycin, and melatonin have been investigated in clinical trials (Table 2). Incorporating the findings of preclinical studies, some of the drugs could target the important molecules of signaling pathways in cardiac repair and recovery of cardiac function. It is remarkable that most of the drugs listed in this review are working through a multi-targeted approach, which directs to multiple molecular targets in different intracellular signaling pathways. For instance, melatonin possesses antioxidant and anti-inflammatory activities post MI^593^; rosuvastatin resists the inflammatory response and excessive fibrosis^594,595^. Besides the usage of drugs, there is also a great possibility to combine drug therapy and classical therapeutic strategies properly. For example, as an adjunct to primary PCI for acute STEMI, the administration of melatonin showed a significant reduction in the infarct size^162^. However, further studies are still needed to explore the intended population, side effects, and optimal dose of drugs^596^.

**Table 2:** Some clinical trials of novel therapeutic strategies for MI

Strategy (drug)	Molecular markers/Signal pathways	Register number	Phase	Estimated/actual enrollment	Status
Ivabradine	PI3K/Akt/mTOR	NCT02446990	III	19102	Completed
Rapamycin/Sirolimus	mTOR	NCT00552669	IV	200	Completed
		NCT04951050	NA	200	Not yet recruiting
Losmapimod	MAPK	NCT02145468	III	3503	Completed
Canakinumab	NLRP3/IL-1β	NCT01327846	III	10066	Completed
		NCT01900600	NA	15	Completed
Colchicine	NLRP3/IL-1β	NCT02551094	III	4745	Completed
		ACTRN12614000093684	III	5522	Completed
		NCT01709981, NCT02594111	IV	280	Completed
		NCT01906749	IV	500	Unknown
		NCT00754819	II & III	80	Completed
		NCT05130892	IV	132	Recruiting
Erythropoietin	MAPK, TGF-β, Wnt, Sonic Hedgehog	NCT00390832	III	138	Completed
		UMIN000005721	NA	600	Completed
		NCT00423020	IV	72	Completed
		NCT00149058	II	124	Unknown
		NCT00524901	II	10	Completed
Estradiol	RhoA/ROCK	NCT00402636	IVIV	502	Completed
Estrogen	RhoA/ROCK	NCT00123539	NA	334	Terminated
Nicorandil	RhoA/ROCK	NCT01396395	IV	402	Completed
Dexmedetomidine	RhoA/ROCK	NCT03095469	I	200	Unknown
Valsartan	TGF-β/SMADs	NCT03309618	NA	36	Completed
Sildenafil	JAK2/STAT3, RhoA/ROCK	NCT01046838	IV	70	Completed
G-CSF	JAK2/STAT3, NF-κB	NCT00596479	NA	50	Completed
		NCT00886509	NA	50	Completed
		NCT00307879	II	20	Terminated
		NCT00043628	II	35	Completed
Methotrexate	NF-κB	NCT01741558	II	80	Completed
Metformin	TLR4	NCT01217307	II & III	380	Completed
Melatonin	Notch, Hippo/YAP	NCT00640094	II	272	Terminated
		NCT03966235	IV	74	Unknown
		NCT00640094	II	272	Terminated
		NCT01172171	II	41	Completed
Fasudil	TGFβ1/TAK1, TGF-β2, TGF-β3, RhoA/ROCK	NCT03753269	IV	600	Not yet recruiting
Statin	PI3K/Akt/ FOXO3a, TGF-β/SMADs, Sonic Hedgehog, RhoA/ROCK/ERK, PI3K/Akt/Nrf2/HO-1	NCT00128024	IV	460	Completed
Hirudin	KEAP1/Nrf2/HO-1	NCT03664180	IV	2856	Recruiting
*Strategy (gene therapy)*					
VEGF-A165 plasmid	VEGF/PI3K/Akt	NCT00135850	I& II	48	Completed
AdGVVEGF121cDNA	VEGF/PI3K/Akt	NCT01174095	I	31	Completed
Endocardial adenovirus VEGF-D gene transfer	VEGF/PI3K/Akt	NCT01002430	I	30	Completed
Bicistronic VEGF-A165/bFGF plasmid	VEGF(bFGF)/PI3K/Akt	NCT00620217	II	52	Completed
*Strategy (Cell therapy)*					
CD34+ cell	PI3K/Akt, Sonic Hedgehog	NCT00300053	II	321	Completed
		NCT03471611	I	20	Active, not recruiting
		NCT01508910	III	291	Completed
CD133+ cell	PI3K/Akt, Wnt	NCT00694642	I& II	28	Completed
		NCT02870933	IV	30	Completed
EPCs	VEGF/PI3K/Akt/eNOS, Dll4/Notch/Hey2, Sonic Hedgehog, Akt/HO-1	NCT00494247	IV	60	Completed
MSCs	PI3K/Akt/mTOR, Wnt/β-catenin, TGF-β/SMADs, Notch, Sonic Hedgehog	NCT00418418	II	60	Unknown

*PI3K/Akt* phosphoinositide-3 kinase/protein kinase B, *MAPK* mitogen-activated protein kinase, NLRP3/IL-1β, *mTOR* mammalian target of rapamycin, *NLRP3/IL-1β* nucleotide-binding domain, leucine-rich-repeat family, pyrin-domain-containing 3/ interleukin-1β, *TGF-β/SMADs* transforming growth factor-β/SMADs, *Wnt* Wingless, *RhoA/ROCK/ERK* Ras homolog family member A/Rho-associated coiled-coil containing protein kinase/extracellular regulated protein kinases, *JAK/STAT* Janus kinase/signal transducer and activator of transcription, *NF-κB* nuclear factor-κB, *TLR4* toll-like receptor 4, *Hippo/YAP*, Hippo/Yes-associated protein, *FOXO3a* forkhead box subfamily O3a, *KEAP1* kelch like ECH-associated protein 1, *Nrf2/HO-1* nuclear factor erythroid derived 2-related factor 2/heme oxygenase-1, *G-CSF* granulocyte colony stimulating factor, *VEGF* vascular endothelial growth factor, *bFGF* basic fibroblast growth factor, *eNOS* endothelial nitric oxide synthase, *EPCs* endothelial progenitor cells, *MSCs* mesenchymal stem cells

Up to now, with the clinical application of gene and cell therapies in MI, some of the current results are encouraging: For example, as the pro-angiogenic growth factor, VEGF binds to VEGF receptors and activates the downstream signaling pathway to promote angiogenesis^40^. Since D. W. Losordo et al. directed myocardial gene transfer of VEGF to treat MI and improved myocardial perfusion in patients in 1998^597^, VEGF gene therapy has been considered an effective therapy for myocardial ischemia, and the long-term safety of gene strategy has been confirmed over a 10 years follow-up in cardiovascular disease^598^; additionally, stem cell therapy for MI is being carried out in many studies, and these adequately powered results promote the development of clinical translation in this field. Further studies also indicated that stem cell therapy might be a potential cardioprotective technique to complement PCI or thrombolytic therapy after AMI^599,600^. Although results of clinical studies on stem cell therapy for myocardial infarction have a certain degree of inconsistency^601,602^, the low immunogenicity, differentiation potential, paracrine action of stem cells could facilitate further studies to demonstrate their clinical efficacy in MI^603,604^. Remarkably, in this review, we list a lot of registered clinical trials (Table 2) which aim to assess the therapeutic potential of gene and stem cell therapy in clinical application, integrated with some basic research findings regarding the influences of therapeutic strategies on cell signaling molecule expression. It is undeniable that, with the gradual development of clinical research, the treatment of coronary heart disease targeting these signaling pathways may be advanced from molecular mechanisms to therapeutic potentials, from bench to bed eventually.

In conclusion, the importance of therapeutic strategies targeting cell signaling molecule expression is emerging which we can not ignore, because it provides us with new evolutional solutions for MI treatment that show potential efficacy in preclinical studies and clinical trials. Moreover, characterization of signaling pathway transduction and regulation in MI development is critical for the determination of targeted therapeutic protocols. Since we have fully combed the roles of signaling pathways in the pathological development and treatment of MI, and the future research direction of myocardial infarction treatment, this information will contribute to the exploration and application of novel therapeutic strategies for MI.
